# Computational strategies to combat COVID-19: useful tools to accelerate SARS-CoV-2 and coronavirus research

**DOI:** 10.1093/bib/bbaa232

**Published:** 2020-11-04

**Authors:** Franziska Hufsky, Kevin Lamkiewicz, Alexandre Almeida, Abdel Aouacheria, Cecilia Arighi, Alex Bateman, Jan Baumbach, Niko Beerenwinkel, Christian Brandt, Marco Cacciabue, Sara Chuguransky, Oliver Drechsel, Robert D Finn, Adrian Fritz, Stephan Fuchs, Georges Hattab, Anne-Christin Hauschild, Dominik Heider, Marie Hoffmann, Martin Hölzer, Stefan Hoops, Lars Kaderali, Ioanna Kalvari, Max von Kleist, Renó Kmiecinski, Denise Kühnert, Gorka Lasso, Pieter Libin, Markus List, Hannah F Löchel, Maria J Martin, Roman Martin, Julian Matschinske, Alice C McHardy, Pedro Mendes, Jaina Mistry, Vincent Navratil, Eric P Nawrocki, Áine Niamh O’Toole, Nancy Ontiveros-Palacios, Anton I Petrov, Guillermo Rangel-Pineros, Nicole Redaschi, Susanne Reimering, Knut Reinert, Alejandro Reyes, Lorna Richardson, David L Robertson, Sepideh Sadegh, Joshua B Singer, Kristof Theys, Chris Upton, Marius Welzel, Lowri Williams, Manja Marz

**Affiliations:** Friedrich-Schiller-University Jena, Germany; Friedrich-Schiller-University Jena, Germany; EMBL-EBI and the Wellcome Sanger Institute, UK; CNRS, France; Biocuration and Literature Access at PIR, USA; Protein Sequence Resources at EMBL-EBI, UK; Technical University of Munich, Germany; Computational Biology at ETH Zurich, Switzerland; Institute of Infectious Disease and Infection Control at Jena University Hospital, Germany; Consejo Nacional de Investigaciones Científicas y Tócnicas (CONICET) working on FMDV virology at the Instituto de Agrobiotecnología y Biología Molecular (IABiMo, INTA-CONICET) and at the Departamento de Ciencias Básicas, Universidad Nacional de Luján (UNLu), Argentina; Pfam and InterPro databases, at the EMBL-EBI, UK; bioinformatics department at the Robert Koch-Institute, Germany; Pfam and MGnify; Computational Biology of Infection Research group of Alice C. McHardy at the Helmholtz Centre for Infection Research, Germany; bioinformatics department at the Robert Koch-Institute, Germany; Bioinformatics Division at Philipps-University Marburg, Germany; Philipps-University Marburg, Germany; Data Science in Biomedicine at the Philipps-University of Marburg, Germany; Freie Universität Berlin, Germany; Friedrich Schiller University Jena, Germany; Biocomplexity Institute and Initiative at the University of Virginia, USA; Bioinformatics and head of the Institute of Bioinformatics at University Medicine Greifswald, Germany; Senior Software Developer; bioinformatics department at the Robert Koch-Institute, Germany; bioinformatics department at the Robert Koch-Institute, Germany; Max Planck Institute for the Science of Human History; Chandran Lab, Albert Einstein College of Medicine, USA; University of Hasselt, Belgium; Technical University of Munich, Germany; Philipps-University Marburg, Germany; EMBL-EBI, UK; Philipps-University Marburg, Germany; Chair of Experimental Bioinformatics at TU Munich, Germany; Computational Biology of Infection Research Lab at the Helmholtz Centre for Infection Research in Braunschweig, Germany; Center for Quantitative Medicine of the University of Connecticut School of Medicine, USA; EMBL-EBI, UK; Bioinformatics and Systems Biology at the Rhône Alpes Bioinformatics core facility, Universitó de Lyon, France; National Center for Biotechnology Information (NCBI); Rambaut group at Edinburgh University, UK; EMBL-EBI, UK; EMBL-EBI, UK; GLOBE Institute in the University of Copenhagen, Denmark; Development of the Swiss-Prot group at the SIB for UniProt and SIB resources that cover viral biology (ViralZone); Computational Biology of Infection Research group of Alice C. McHardy at the Helmholtz Centre for Infection Research; Freie Universität Berlin, Germany; Universidad de los Andes, Colombia; Sequence Families team at EMBL-EBI, UK; MRC-University of Glasgow Centre for Virus Research, UK; Chair of Experimental Bioinformatics at Technical University of Munich, Germany; MRC-University of Glasgow Centre for Virus Research, Glasgow, Scotland, UK; Rega institute of the University of Leuven, Belgium; Department of Biochemistry and Microbiology, University of Victoria, Canada; Philipps-University Marburg, Germany; Pfam and InterPro databases, at EMBL-EBI, UK; Friedrich Schiller University Jena, Germany

**Keywords:** virus bioinformatics, SARS-CoV-2, sequencing, epidemiology, drug design, tools

## Abstract

SARS-CoV-2 (severe acute respiratory syndrome coronavirus 2) is a novel virus of the family *Coronaviridae*. The virus causes the infectious disease COVID-19. The biology of coronaviruses has been studied for many years. However, bioinformatics tools designed explicitly for SARS-CoV-2 have only recently been developed as a rapid reaction to the need for fast detection, understanding and treatment of COVID-19. To control the ongoing COVID-19 pandemic, it is of utmost importance to get insight into the evolution and pathogenesis of the virus. In this review, we cover bioinformatics workflows and tools for the routine detection of SARS-CoV-2 infection, the reliable analysis of sequencing data, the tracking of the COVID-19 pandemic and evaluation of containment measures, the study of coronavirus evolution, the discovery of potential drug targets and development of therapeutic strategies. For each tool, we briefly describe its use case and how it advances research specifically for SARS-CoV-2. All tools are free to use and available online, either through web applications or public code repositories. **Contact:**evbc@unj-jena.de

## Introduction

On 31 December 2019, the Wuhan Municipal Health Commission reported several cases of pneumonia in Wuhan (China) to the World Health Organization (https://www.who.int/csr/don/05-january-2020-pneumonia-of-unkown-cause-china/en/). The cause of these cases was a previously unknown coronavirus, now known as severe acute respiratory syndrome coronavirus 2 (SARS-CoV-2), which can manifest itself in the disease named COVID-19. At the time of writing (22 July 2020), nearly 15 million cases were reported worldwide, with over 600 000 deaths (https://www.who.int/docs/default-source/coronaviruse/situation-reports/20200722-covid-19-sitrep-184.pdf). The group of *Coronaviridae* includes viruses with very long RNA genomes up to 33 000 nucleotides. SARS-CoV-2 belongs to the *Sarbecovirus* subgenus (genus: *Betacoronavirus*) and has a genome of approximately 30 000 nucleotides [119]. In line with other members of *Coronaviridae*, SARS-CoV-2 has four main structural proteins: spike (S), envelope (E), membrane (M) and nucleocapsid (N). Further, several nonstructural proteins are encoded in the pp1a and pp1ab polyproteins, which are essential for viral replication [119]. SARS-CoV-2 seems to use the human receptor ACE2 as its main entry [34], which has been observed for other *Sarbecoviruses* as well [32, 55]. The binding domains for ACE2 are located on the spike proteins, which further contain a novel furin cleavage site, associated with increased pathogenicity and transmission potential [46, 66, 95, 112].

Although SARS-CoV-2 has a lower mutation rate than most RNA viruses, mutations certainly accumulate and result in genomic diversity both between and within individual infected patients. Genetic heterogeneity enables viral adaptation to different hosts and different environments within hosts and is often associated with disease progression, drug resistance and treatment outcome.

In light of the COVID-19 pandemic, there has been a rapid increase in SARS-CoV-2-related research. It will be critical to get insight into the evolution and pathogenesis of the virus in order to control this pandemic. Researchers around the world are investigating SARS-CoV-2 sequence evolution on genome and protein level, tracking the pandemic using phylodynamic and epidemiological models and examining potential drug targets. Laboratories are sharing SARS-CoV-2-related data with unprecedented speed. In light of this sheer amount of data, many fundamental questions in SARS-CoV-2 research can only be tackled with the help of bioinformaticians. Adequate analysis of these data has the potential to boost discovery and inform both fundamental and applied science, in addition to public health initiatives.

SARS-CoV-2 is an entirely novel pathogen, and in light of the pandemic requiring a swift response to research and public health-related questions, the natural first approach is to repurpose existing methods and resources. Simultaneously, the outbreak has had a huge impact on virus bioinformatics tools that have been developed recently and it is important to understand which tools are applicable to coronaviruses and which have been customized to address research questions related to SARS-CoV-2.

In this review, we cover bioinformatics workflows and tools (see Table 1) starting with the routine detection of SARS-CoV-2 infection, the reliable analysis of sequencing data, the tracking of the COVID-19 pandemic, the study of coronavirus evolution, up to the detection of potential drug targets and development of therapeutic strategies. All tools have either been developed explicitly for SARS-CoV-2 research, have been extended or adapted to coronaviruses or are of particular importance to study SARS-CoV-2 epidemiology and pathogenesis.

**Table 1:** Bioinformatics tools accelerating SARS-CoV-2 research. Overview of all workflows and tools covered in this review. All tools are free to use and available online. A list of these and further tools can be found on the website of the European Virus Bioinformatics Center (EVBC): http://evbc.uni-jena.de/tools/coronavirus-tools/

**Tool**	**Advancing SARS-CoV-2 research by**	**License**	**Link(s)**
Detection and annotation			
PriSeT	computing SARS-CoV-2 specific primers for RT-PCR tests	GPLv3	https://github.com/mariehoffmann/PriSeT
CoVPipe	reproducible, reliable and fast analysis of NGS data	GPLv3	https://gitlab.com/RKIBioinformatics Pipelines/ncov_minipipe
poreCov	reducing time-consuming bioinformatic bottlenecks in processing sequencing runs	GPLv3	https://github.com/replikation/poreCov
VADR	validation and annotation of SARS-CoV-2 sequences	public domain	https://github.com/nawrockie/vadr
V-Pipe	reproducible NGS-based, end-to-end analysis of genomic diversity in intra-host virus populations	APLv2	https://cbg-ethz.github.io/V-pipe/ https://github.com/cbg-ethz/V-pipe
Haploflow	detection and full-length reconstruction of multi-strain infections	APLv2	https://github.com/hzi-bifo/Haploflow
VIRify	identifying viruses in clinical samples	APLv2	https://github.com/EBI-Metagenomics/emg-viral-pipeline
VBRC genome analysis tools	visualizing differences between coronavirus sequences at different levels of resolution	GPLv3	https://www.4virology.net
VIRULIGN	fast, codon-correct multiple sequence alignment and annotation of virus genomes	GPLv2	https://github.com/rega-cev/virulign
Rfam COVID-19	annotating structured RNAs in coronavirus sequences and predicting secondary structures	CC0	https://rfam.org/covid-19
UniProt COVID-19	providing latest knowledge on proteins relevant to the disease for virus and host	CC BY 4.0	https://covid-19.uniprot.org/
Pfam	protein detection and annotation for outbreak tracking and studying evolution	CC0	https://pfam.xfam.org
Tracking, epidemiology and evolution			
Covidex	fast and accurate subtypification of SARS-CoV-2 genomes	GPLv3	https://sourceforge.net/projects/covidex https://cacciabue.shinyapps.io/shiny2/
Pangolin	assigning a global lineage to query genomes	GPLv3	https://pangolin.cog-uk.io/ https://github.com/hCoV-2019/pangolin/
BEAST 2	understanding geographical origin and evolutionary and transmission dynamics	LGPL	https://www.beast2.org/
Phylogeographic reconstruction	studying the global spread of the pandemic with particular focus on air transportation data	APLv2	https://github.com/hzi-bifo/Phylogeography_Paper
COPASI	modelling the dynamics of the epidemic and effect of interventions	Artistic License 2.0	http://copasi.org/ https://github.com/copasi
COVIDSIM	analysing effects of contact reduction measures and guide political decision-making	—	http://www.kaderali.org:3838/covidsim
CoV-GLUE	tracking changes accumulating in the SARS-CoV-2 genome	(AGPLv3)^a^	http://cov-glue.cvr.gla.ac.uk/
PoSeiDon	detection of positive selection in protein-coding genes	MIT License	https://github.com/hoelzer/poseidon
Drug design			
VirHostNet	understanding molecular mechanisms underlying virus replication and pathogenesis	—^b^	http://virhostnet.prabi.fr/
CORDITE	carrying out meta-analyses on potential drugs and identifying potential drug candidates for clinical trials	CC BY-ND	https://cordite.mathematik.uni-marburg.de
CoVex	identifying already approved drugs that could be repurposed to treat COVID-19	— ^c^	https://exbio.wzw.tum.de/covex/
P-HIPSTer	enabling the discovery of PPIs commonly employed within the coronavirus family and PPIs associated with their pathogenicity	— ^d^	http://www.phipster.org/

^a^License of the underlying software system GLUE; ^b^All data open access; ^c^Source code available upon request; ^d^Predictions available upon request.

## Detection and annotation

The routine detection method for SARS-CoV-2 is a real-time quantitative reverse transcriptase polymerase chain reaction (qRT-PCR). The test is based on the detection of two nucleotide sequences: the virus envelope (E) gene and the gene for the RNA-dependent RNA polymerase (RdRp) [11]. Specificity (exclusion of false positives) and sensitivity (exclusion of false negatives) are two of the most important quality criteria for the validity of diagnostic tests. To ensure unique identification of SARS-CoV-2 and avoid false-negative and false-positive detection, the computation of SARS-CoV-2-specific primers is required. A new set of primers might be required if the specificity or sensitivity of the qRT-PCR test changes due to mutations in the SARS-CoV-2 genome or related coronavirus genomes (see PriSeT).

Besides qRT-PCR, genome analysis plays a crucial role in public health responses, including epidemiological efforts to track and contain the outbreak (see Tracking, epidemiology and evolution). The genome sequence of SARS-CoV-2 was rapidly determined and shared on GenBank (MN908947.3). It is annotated based on sequence similarity to other coronaviruses. Next-generation sequencing (NGS) can be used to assess the genomic diversity of the virus. Regular sequencing from clinical cases is useful, for example, to monitor for mutations that might affect the qRT-PCR test (see CoVPipe, V-Pipe). To reliably derive intra-host diversity estimates from deep sequencing data is challenging since most variants occur at low frequencies in the virus population and amplification and sequencing errors confound their detection. Multiple related viral strains (haplotypes) are hard to resolve but may be critical for the choice of therapy (see Haploflow, V-Pipe).

The SARS-CoV-2 nanopore sequencing protocol has been developed and optimized by the ARTIC network [78], which has extensive experience and expertise in deploying this technology in the sequencing and surveillance of outbreaks, including Zika and Ebola [79]. Nanopore sequencing is used to quickly generate high-accuracy genomes of SARS-CoV-2 and track both transmission of COVID-19 and viral evolution over time (see poreCov).

In addition to amplicon-based sequencing approaches, metagenomic/-transcriptomic sequencing offers the ability to identify the primary pathogen and additional infections that may be present [70]. It can be used to identify coronaviruses in clinical and environmental samples, e.g. from human Bronchoalveolar lavage fluid (see VIRify). SARS-CoV-2 genomic traces in human faecal metagenomes from before the pandemic support the hypothesis of a possible presence of a most recent common ancestor of SARS-CoV-2 in the human population before the outbreak of the current pandemic, possibly in an inactive non-virulent form [81]. Further, metagenomics helps to check sequence divergence as the virus could undergo mutation and recombination with other human coronaviruses.

To help fight the COVID-19 pandemic, it is essential to make high-quality SARS-CoV-2 genome sequence data and metadata available through open databases either via a data-access agreement (e.g. GISAID, https://www.gisaid.org/) or without restrictions (e.g. GenBank, https://submit.ncbi.nlm.nih.gov/sarscov2/). On GISAID (Global Initiative on Sharing All Influenza Data), laboratories around the world have shared viral genome sequence data with unprecedented speed (>71 000 SARS-CoV-2 genomic sequences on 23 July 2020). We encourage researchers to submit genome sequences to public databases that do not impose limitations on the sharing and use of the genomic sequences. NCBI offers a new streamlined submission process for SARS-CoV-2 data (https://ncbiinsights.ncbi.nlm.nih.gov/2020/04/09/sars-cov2-data-streamlined-submission-rapid-turnaround/).

Several bioinformatics tools have been developed for the detection and annotation of SARS-CoV-2 genomes (see VADR, V-Pipe, VIRify, VBRC tools, VIRULIGN). Comparative genomics helps to detect differences to other coronaviruses, e.g. SARS-CoV-1, which might affect the functionality and pathogenesis of the virus.

Aside from coding sequences and proteins, the identification of conserved functional RNA secondary structures (see Rfam) is essential to understanding the molecular mechanisms of the virus life cycle [63, 91]. Coronaviruses are known to have highly structured, conserved untranslated regions, which harbour *cis*-regulatory RNA secondary structure, controlling viral replication and translation, and even small changes in these structures reduce the viral load drastically [25, 61, 120].

Studying viral genomic diversity and the evolution of coding and non-coding sequences (see UniProt, Pfam, Rfam) is important for a better understanding of the evolution and epidemiology of SARS-CoV-2 (see **Tracking, epidemiology and evolution**) and the molecular mechanisms underlying COVID-19 pathogenesis (see **Drug design**).

### PriSeT: Primer Search Tool

PriSeT [35] is a software tool that identifies chemically suitable PCR primers in a reference data set. The reference data set can be a FASTA file of complete genomes or a set of short regions. It is optimized for metabarcoding experiments where species are identified from an environmental sample based on a barcode—a relatively short region from the genome. The most frequently applied type of PCR for such experiments is the paired-end PCR—two different primer sequences are chosen to be complementary to the template and located within an offset range. The region in between is the amplicon or barcode and will be matched against the reference database to resolve operational taxonomic units to organisms. The precise constraint ranges can be adjusted by the user.

**Figure 1:**
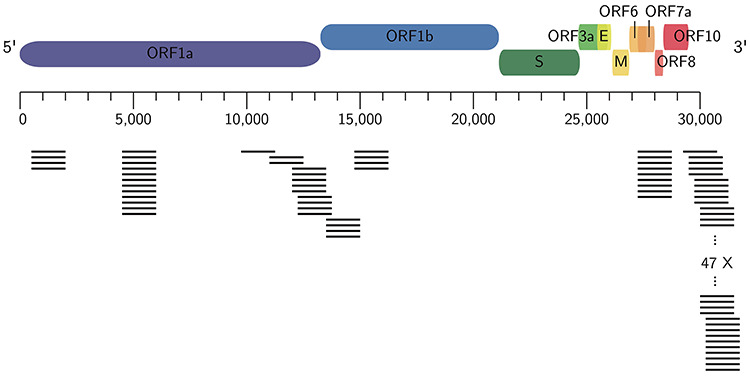
**SARS-CoV-2-specific primers computed with PriSeT.** Approximate amplicon locations of *de novo* computed primer pairs for SARS-CoV-2 with no co-occurrences in other genomes in GenBank (on 3 April 2020).

SARS-CoV-2 tests typically use mucus from the nose or throat that undergo a metabarcoding analysis. For DNA amplification RT-PCR is applied, which has more stringent requirements for the primer sequences and the DNA product length. Figure 1 shows the approximate locations of *in silico* transcripts of 114 primer pairs computed by PriSeT on 19 SARS-CoV-2 genomes with recommended RT-PCR settings [35]. The corresponding primer pairs have no co-occurrences in other coronaviruses. An additional online search on NCBI’s GenBank confirmed that they also have no matches in any other sequences that are not assigned to SARS-CoV-2. A list of SARS-CoV-2-specific primer pairs computed on 19 SARS-CoV-2 genomes is available on ResearchGate (https://www.researchgate.net/publication/340418344_Primer_ pairs_for_detection_of_SARS-CoV-2_via_RT-PCR).

The computation of SARS-CoV-2-specific primers will help to design RT-PCR tests, since the resulting barcodes serve as unique identifiers for SARS-CoV-2 and avoid false-negative and false-positive identifications.

PriSeT is hosted on GitHub under the GNU General Public License v3.0 (GPLv3): https://github.com/mariehoffmann/PriSeT.

### CoVPipe: amplicon-based genome reconstruction

CoVPipe is a highly optimized and fully automated workflow for the reference-based reconstruction of SARS-CoV-2 genomes based on next-generation amplicon sequencing data using CleanPlex^®^ SARS-CoV-2 panels (Paragon Genomics, Hayward, CA, USA) from swab samples. The pipeline applies read classification, clipping of raw reads to remove terminal PCR primer sequences or primer hybrids as well as Illumina adapters and low-quality bases. The processed reads are then aligned to a given reference sequence using BWA-MEM [54]. Resulting BAM files are evaluated to report mapping quality measurements like coverage, read depth and insert size (bedtools v2.27 and samtools v1.3). Variants are called using GATK (v4.1) [64] and filtered following best practices of GATK. Finally, different consensus sequences can be created using different masking methods. Additionally, detailed information such as coverage, genomic localization and effect on respective gene products are reported for each variant site.

The pipeline is designed for reproducibility and scalability in order to ensure reliable and fast data analysis of SARS-CoV-2 data. The workflow itself is implemented using Snakemake [48], which provides advanced job balancing and input/output control mechanisms, and uses conda [28] to provide well-defined and harmonized software environments.

CoVPipe is available via GitLab under GPLv3: https://gitlab.com/RKIBioinformaticsPipelines/ ncov_minipipe.

### poreCov: rapid sample analysis for nanopore sequencing

Nanopore workflows were previously used in other outbreak situations, e.g. Zika, Ebola, Yellow Fever, Swine Flu, and can deliver a consensus viral genome after approximately 7 hours (https://nanoporetech.com/about-us/news/novel-coronavirus-covid-19-information-and-updates). The ARTIC network provides all the necessary information, tools and protocols to assist groups in sequencing the coronavirus via nanopore sequencing (https://artic.network/ncov-2019). These protocols utilize a multiplex PCR approach to amplify the virus directly from clinical samples, followed by sequencing and bioinformatic steps to assemble the data (https://artic.network/ncov-2019/ncov2019-bioinformatics-sop.html). Due to the small viral genome, up to 24 samples can be sequenced at the same time. Rapid sample analysis is, therefore, of particular interest.

**Figure 2:**
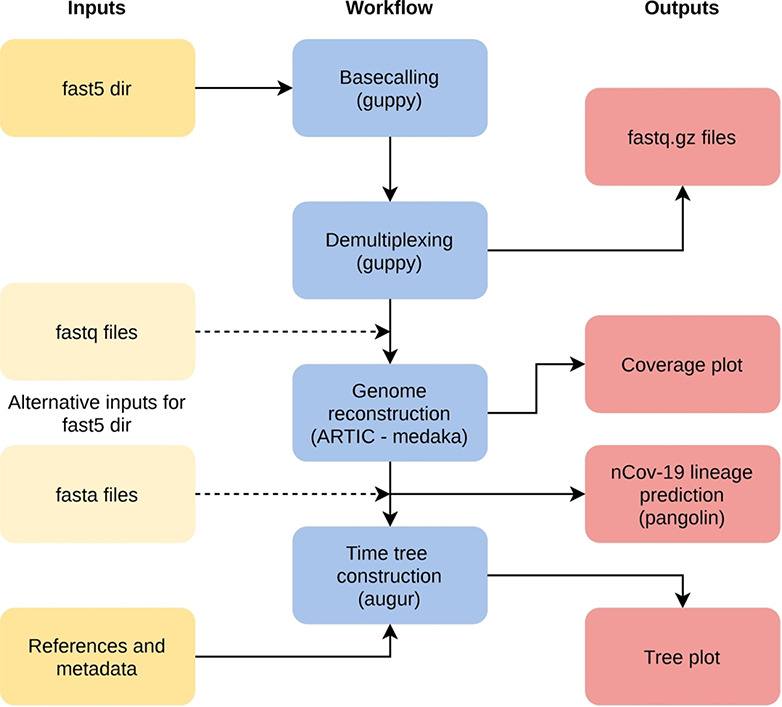
**Simplified overview of the poreCov workflow.** The individual workflow steps (blue) are executed automatically depending on the input (yellow). Instead of using raw nanopore fast5 files, fastq files or complete SARS-CoV-2 genomes can be used as an alternative input. If reference genomes and location/times are added, a time tree is additionally constructed.

The workflow poreCov is implemented in nextflow [100] for full parallelization of the workload and stable sample processing (see Figure 2). poreCov generates all necessary results and information before scientists continue to analyze their genomes or make them public on, e.g. GISAID or ENA / NCBI. The workflow carries out all necessary steps from basecalling to assembly depending on the user input, followed by lineage prediction of each genome using Pangolin (see below). Furthermore, read coverage plots are provided for each genome to assess the amplification quality of the multiplex PCR. In addition, poreCov includes a quick-time, tree-based analysis of the inputs against reference sequences using augur (https://github.com/nextstrain/augur) and toytree (https://github.com/eaton-lab/toytree) for visualization. poreCov supports scientists in their SARS-CoV-2 research by reducing the time-consuming bioinformatic bottlenecks in processing dozens of SARS-CoV-2 sequencing runs.

All tools are provided via ‘containers’ (pre-build and stored on docker hub) to generate a reproducible workflow in various working environments. poreCov is available on GitHub under GPLv3: https://github.com/replikation/poreCov.

### VADR: SARS-CoV-2 genome annotation and validation

VADR validates and annotates viral sequences based on models built from reference sequences [85]. Coronavirus models, based on NCBI RefSeq [73] entries, including one for SARS-CoV-2 (NC_045512.2), are available for analyzing coronavirus sequences. VADR computes an alignment of each incoming sequence against the RefSeq and uses it to map the RefSeq features, which include protein coding sequences (CDS), genes, mature peptides (mat_peptide) and structural RNA (stem_loop) features. The ORF1ab polyprotein CDS involves a programmed ribosomal frameshift, which VADR is capable of properly annotating. The tool identifies and outputs information about more than 40 types of problems with sequences, such as early stop codons in CDS, and has been in use by GenBank for screening and annotating incoming SARS-CoV-2 sequence submissions since March 2020. VADR (v1.1) includes heuristics for accelerating annotation and for dealing with stretches of ambiguous N nucleotides, that were specifically added for SARS-CoV-2 analysis.

VADR helps advance SARS-CoV-2 research by standardizing the annotation of SARS-CoV-2 sequences deposited in GenBank and other databases and by allowing researchers to fully annotate and screen their sequences for errors due to misassembly or other problems.

VADR is available via GitHub (public domain): https://github.com/nawrockie/vadr, including specific instructions for use on SARS-CoV-2 sequences (https://github.com/nawrockie/vadr/wiki/Coronavirus-annotation).

### V-Pipe: calling single-nucleotide variants and viral haplotypes

V-pipe [77] is a bioinformatics pipeline that integrates various computational tools for the analysis of viral high-throughput sequencing data. It supports the reproducible end-to-end analysis of intra-host NGS data, including quality control, read mapping and alignment and inference of viral genomic diversity on the level of both single-nucleotide variants (SNVs) and long-range viral haplotypes. V-pipe uses the workflow management system Snakemake [48] to organize the order of required computational steps, and it supports cluster computing environments. It is easy to use from the command line, and conda [28] environments facilitate installation. V-pipe’s modular architecture allows users to design their pipelines and developers to test their tools in a defined environment, enabling best practices for viral bioinformatics.

A recent release of V-pipe addresses specifically the analysis of SARS-CoV-2 sequencing data. It uses the strain NC_045512 (GenBank: MN908947.3) as the default for read mapping and reporting of genetic variants, and it includes several improvements, for example, for calling single-nucleotide variants. Also, V-pipe can generate a comprehensive and intuitive visualization of the detected genomic variation in the context of various annotations of the SARS-CoV-2 genome. This summary of the output can help to generate diagnostic reports based on viral genomic data.

V-pipe is an SIB resource (https://www.sib.swiss/research-infrastructure/database-software-tools/sib-resources) and available via GitHub under the Apache License 2.0 (APLv2): https://github.com/cbg-ethz/V-pipe. Users are supported through the website (https://cbg-ethz.github.io/V-pipe/), tutorials, videos, a mailing list and the dedicated wiki pages of the GitHub repository.

### Haploflow: Multi-strain aware *de novo* assembly

Viral infections often include multiple related viral strains [113], either due to co-infection or within-host evolution. These strains - haplotypes - may vary in phenotype due to certain, strain-specific genetic properties [51]. It is not entirely clear yet whether SARS-CoV-2 has a tendency for multiple infections, though there are indications that co-infections with other Coronaviruses do occur [59]. Most assemblers struggle with resolving complete viral haplotypes, even though these may be critical for the choice of therapy. Haploflow is a novel, de Bruijn graph-based assembler for the *de novo*, strain-resolved assembly of viruses that is able to rapidly resolve differences up to a base-pair level between two viral strains. Haploflow will help advance SARS-CoV-2 research by enabling the detection and full-length reconstruction of SARS-CoV-2 multi-strain infections.

Haploflow is available on GitHub under APLv2: https://github.com/hzi-bifo/Haploflow

### VIRify: Annotation of viruses in meta-omic data

VIRify is a recently developed generic pipeline for the detection, annotation and taxonomic classification of viral and phage contigs in metagenomic and metatranscriptomic assemblies. This pipeline is part of the repertoire of analysis services offered by MGnify [69]. VIRify’s taxonomic classification relies on the detection of taxon-specific profile hidden Markov models (HMMs), built upon a set of 22,014 orthologous protein domains and referred to as ViPhOGs. Included in this profile HMM database are 139 models that serve as specific markers for taxa within the *Coronaviridae* family.

**Figure 3:**
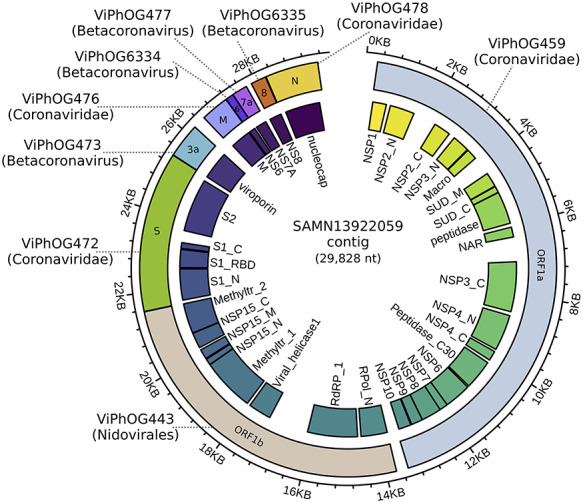
**Sequence reads from a human lung metatranscriptome** (sample accession: SAMN13922059) were first quality-filtered using TrimGalore v0.6.0 and subsequently assembled using MEGAHIT v1.1.3 [53] with default parameters. The resulting metatranscriptome assembly was processed through the VIRify pipeline. Based on the hits against the ViPhOG database, a 29 kb contig was classified as *Coronaviridae*. Functional protein domain annotations (inner track) were assigned by an hmmsearch v3.1b2 against coronavirus models in Pfam. The image was created with circlize [29] and polished with Inkscape.

Here, we show the applicability of VIRify on the assembly of a metatranscriptomic dataset from a human Bronchoalveolar lavage fluid. Within this assembly, a 29 kb contig was classified by VIRify as belonging to the *Coronaviridae* family (see Figure 3). This shows the utility of the VIRify pipeline, used in isolation from MGnify, for studying coronaviruses in the human respiratory microbiome.

VIRify can be used for the identification of coronaviruses in clinical and environmental samples. Due to the intrinsic differences between metatranscriptomes and metagenomes, additional considerations regarding quality control, assembly, post-processing and classification have to be kept in mind (for details, see https://github.com/EBI-Metagenomics/emg-viral-pipeline).

VIRify is available via GitHub under APLv2: https://github.com/EBI-Metagenomics/emg-viral-pipeline.

### Genome analysis tools by VBRC

The Viral Bioinformatics Research Centre (VBRC) is a mature resource built specifically for virologists to facilitate the comparative analysis of viral genomes. Within VBRC, a MySQL database created from GenBank files supports numerous analysis tools. The curated database is accessed through Virus Orthologous Clusters [16], a powerful, but easy-to-use database GUI. Base-By-Base [9, 33, 102] is a tool for generating, visualizing and editing multiple sequence alignments. It can compare genomes, genes or proteins via alignments and plots. Users can add comments to sequences and save alignments to a local computer. Viral Genome Organizer [105] visualizes and compares the organization of genes within multiple complete viral genomes. The tool allows the user to export protein or DNA sequences and can display START/STOP codons for 6-frames as well as open reading frames and other user-defined results. If genomes are loaded from the database, it can display shared orthologs. Genome Annotation Transfer Utility [98] is a tool for annotating genomes using information from a reference genome. It provides for interactive annotation, automatically annotating genes that are very similar to the reference virus but leaving others for a human decision.

**Figure 4:**
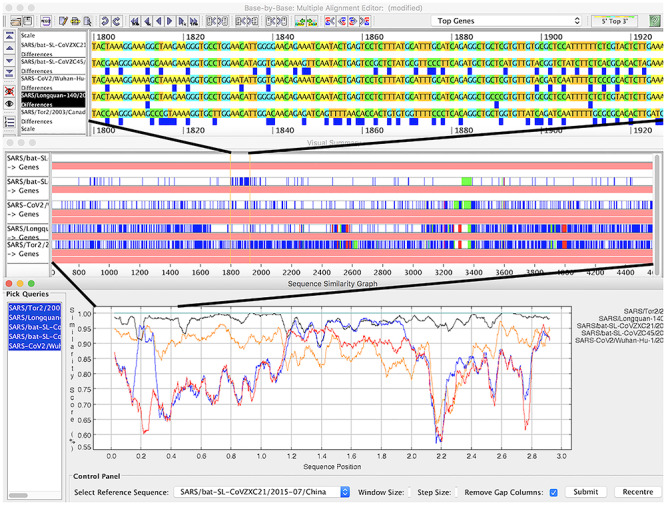
**A region of recombination in coronavirus genomes at three levels of resolution in Base-By-Base.** Top panel: aligned genomes; blue boxes show differences compared to top sequence in alignment. Middle panel: summary view showing differences and indels compared top sequence. Bottom panel: similarity plot comparing five genomes.

The VBRC was developed for dsDNA viruses but has been adapted for coronaviruses. SARS-CoV-2 and closely related viruses have been added to the database. VBRC tools will help to visualize differences between coronavirus sequences at different levels of resolution (see Figure 4).

VBRC is available via https://www.4virology.net; all tools are published under GPLv3.

### VIRULIGN: Codon-correct multiple sequence alignments

VIRULIGN was developed for fast, codon-correct multiple sequence alignment and annotation of virus genomes, guided by a reference sequence [58]. A codon-aware alignment is essential for studying the evolution of coding nucleotide sequences to aid vaccine and antiviral development [12], to understand the emergence of drug resistance [72] and to quantify epidemiological potential [76]. [99] have shown that a representative and curated annotation of open reading frames and proteins is essential to study emerging pathogens. To this end, a SARS-CoV-2 reference sequence and genome annotation have been added to VIRULIGN, based on the first available genome sequence [119], covering all reading frames and proteins.

VIRULIGN is easy to install, enabling scientists to perform large-scale analyses on their local computational infrastructure. VIRULIGN is particularly well suited to study the rapidly growing number of SARS-CoV-2 genomes made available [80], due to its efficient alignment algorithm that has linear computational complexity with respect to the number of sequences studied. Furthermore, VIRULIGN’s flexible output formats (e.g. CSV file with headers corresponding to the genome annotation) facilitate its integration into analysis workflows, lowering the threshold for scientists to deliver advanced bioinformatics pipelines [13, 57] and databases [56], that are necessary to track the COVID-19 pandemic.

VIRULIGN is available via GitHub under the the GNU General Public License v2.0 (GPLv2): https://github.com/rega-cev/virulign.

### Rfam COVID-19 resources: coronavirus-specific RNA families

Rfam [40] is a database of RNA families that hosts curated multiple sequence alignments and covariance models. To facilitate the analysis of Coronavirus sequences, Rfam produced a special release 14.2 with ten new families representing the entire 5’ and 3’ untranslated regions (UTRs) from *Alpha-*, *Beta-*, *Gamma-* and *Deltacoronaviruses*. A specialized set of *Sarbecovirus* models is also provided, which includes SARS-CoV-1 and SARS-CoV-2 sequences. The families are based on a set of high-quality whole genome alignments that have been reviewed by expert virologists. In addition, Rfam now contains a revised set of non-UTR Coronavirus structured RNAs, such as the frameshift stimulating element, s2m RNA, and the 3’ UTR pseudoknot.

**Figure 5:**
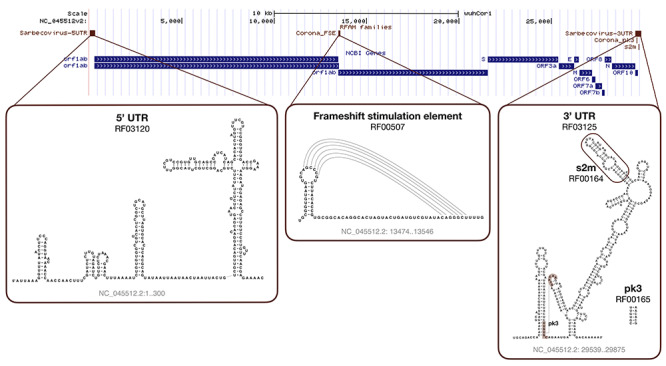
**SARS-CoV-2 Rfam secondary structure predictions.** The sequence is based on the NC_045512.2 RefSeq entry displayed with the wuhCor1 UCSC Genome Browser alongside the NCBI Genes track.

The new Rfam families can be used in conjunction with the Infernal software [71] to annotate structured RNAs in Coronavirus sequences and predict their secondary structure (see Figure 5). Table 2 shows the results for the SARS-CoV-2 RefSeq entry (NC_045512.2). In addition, the online Rfam sequence search enables users to scan genomic sequences and find the RNA elements.

**Table 2:** Rfam version 14.2 matches to the SARS-CoV-2 RefSeq entry NC_045512.2

**RefSeq coordinates**	**Rfam accession**	**Rfam ID**	**Rfam description**	**Comment**
NC_045512.2/1-299	RF03120	Sarbecovirus-5UTR	Sarbecovirus 5’ UTR	See Rfam family RF03117 for Betacoronavirus 5’ UTR.
NC_045512.2/13,469-13,550	RF00507	Corona_FSE	Coronavirus frameshifting stimulation element	
NC_045512.2/29,536-29,870	RF03125	Sarbecovirus-3UTR	Sarbecovirus 3’ UTR	See Rfam family RF03122 for Betacoronavirus 3’ UTR.
NC_045512.2/29,603-29,662	RF00164	Corona_pk3	Coronavirus 3’ UTR pseudoknot	The family annotates the pseudoknot found in the 3’ UTR (RF03120).
NC_045512.2/29,727-29,769	RF00165	s2m	Coronavirus 3’ stem-loop II-like motif (s2m)	The family is a subset of the 3’ UTR model (RF03120) that corresponds to the PDB:1XJR 3D structure from SARS-CoV-1.

The Coronavirus Rfam families are available freely available under the Creative Commons Zero (CC0) licence at https://rfam.org/covid-19.

### UniProt COVID-19 protein portal: rapid access to protein information

UniProt [104] has recognized the urgency of annotating and providing access to the latest information on proteins relevant to the disease for both the virus and human host. In response, the COVID-19 UniProt portal provides early pre-release access to (i) SARS-CoV-2 annotated protein sequences, (ii) closest SARS proteins from SARS 2003, (iii) human proteins relevant to the biology of viral infection, like receptors and enzymes, (iv) ProtVista [115] visualization of sequence features for each protein, (v) links to sequence analysis tools, (vi) access to collated community-contributed publications relevant to COVID-19, as well as (vii) links to relevant resources.

The COVID-19 portal enables community crowdsourcing of publications via the “Add a publication” feature within any entry. Thus, the community can assist in associating new or missing publications to relevant UniProt entries. ORCID is used as a mechanism to validate user credentials as well as recognition for contribution. Ten publication submissions have been received so far, contributing to our understanding of the virus biology. The COVID-19 UniProt portal advances SARS-CoV-2 research by providing latest knowledge on proteins relevant to the disease for both the virus and human host.

The COVID-19 UniProt portal is available under the Creative Commons Attribution License (CC BY 4.0) via https://covid-19.uniprot.org/. UniProt also hosted webinars to describe the portal (https://www.youtube.com/watch?v=EY69TjnVhRs) and publication submission system (https://www.youtube.com/watch?v=sOPZHLtQK9k).

### Pfam protein families database

The Pfam protein families database is widely used in the field of molecular biology for large-scale functional annotation of proteins [17]. The latest release of Pfam, version 33.1, contains an updated set of models that comprehensively cover the proteins encoded by SARS-CoV-2 (see Table 3). The only SARS-CoV-2 protein that lacks a match is Orf10, a small putative protein found at the 3’-end of the SARS-CoV-2 genome, which appears to lack similarity to any other sequence in UniProtKB (https://covid-19.uniprot.org/). The Pfam profile hidden Markov model (HMM) library in combination with the HMMER software [15] facilitates rapid search and annotation of coronaviruses and can be used to generate multiple sequence alignments that allow the identification of mutations and clusters of related sequences, particularly useful for outbreak tracking and studying the evolution of coronaviruses.

**Table 3:** Pfam version 33.1 matches to the proteome of SARS-CoV-2 found in UniProtKB

**Uniprot accession ID**	**Gene name**	**Pfam accession**	**Pfam ID**	**Pfam description**
sp$\mid $P0DTC1$\mid $R1A_SARS2	ORF1ab	PF11501	bCoV_NSP1	Betacoronavirus replicase NSP1
		PF19211	CoV_NSP2_N	Coronavirus replicase NSP2, N-terminal
		PF19212	CoV_NSP2_C	Coronavirus replicase NSP2, C-terminal
		PF12379	bCoV_NSP3_N	Betacoronavirus replicase NSP3, N-terminal
		PF01661	Macro	Macro domain
		PF11633	bCoV_SUD_M	Betacoronavirus single-stranded poly(A)-binding domain
		PF12124	bCoV_SUD_C	Betacoronavirus SUD-C domain
		PF08715	CoV_peptidase	Coronavirus papain-like peptidase
		PF16251	bCoV_NAR	Betacoronavirus nucleic acid-binding (NAR)
		PF19218	CoV_NSP3_C	Coronavirus replicase NSP3, C-terminal
		PF19217	CoV_NSP4_N	Coronavirus replicase NSP4, N-terminal
		PF16348	CoV_NSP4_C	Coronavirus replicase NSP4, C-terminal
		PF05409	Peptidase_C30	Coronavirus endopeptidase C30
		PF19213	CoV_NSP6	Coronavirus replicase NSP6
		PF08716	CoV_NSP7	Coronavirus replicase NSP7
		PF08717	CoV_NSP8	Coronavirus replicase NSP8
		PF08710	CoV_NSP9	Coronavirus replicase NSP9
		PF09401	CoV_NSP10	Coronavirus RNA synthesis protein NSP10
sp$\mid $P0DTC2$\mid $SPIKE_SARS2	S	PF16451	bCoV_S1_N	Betacoronavirus-like spike glycoprotein S1, N-terminal
		PF09408	bCoV_S1_RBD	Betacoronavirus spike glycoprotein S1, receptor binding
		PF19209	CoV_S1_C	Coronavirus spike glycoprotein S1, C-terminal
		PF01601	CoV_S2	Coronavirus spike glycoprotein S2
sp$\mid $P0DTC3$\mid $AP3A_SARS2	ORF3a	PF11289	bCoV_viroporin	Betacoronavirus viroporin
sp$\mid $P0DTC4$\mid $VEMP_SARS2	E	PF02723	CoV_E	Coronavirus small envelope protein E
sp$\mid $P0DTC5$\mid $VME1_SARS2	M	PF01635	CoV_M	Coronavirus M matrix/glycoprotein
sp$\mid $P0DTC6$\mid $NS6_SARS2	ORF6	PF12133	bCoV_NS6	Betacoronavirus NS6 protein
sp$\mid $P0DTC7$\mid $NS7A_SARS2	ORF7a	PF08779	bCoV_NS7A	Betacoronavirus NS7A protein
sp$\mid $P0DTD8$\mid $NS7B_SARS	ORF7b	PF11395	bCoV_NS7B	Betacoronavirus NS7B protein
sp$\mid $P0DTC8$\mid $NS8_SARS2	ORF8	PF12093	bCoV_NS8	Betacoronavirus NS8 protein
sp$\mid $P0DTC9$\mid $NCAP_SARS2	N	PF00937	CoV_nucleocap	Coronavirus nucleocapsid
sp$\mid $P0DTD2$\mid $ORF9B_SARS2	ORF9b	PF09399	bCoV_lipid_BD	Betacoronavirus lipid-binding protein
sp$\mid $P0DTD3$\mid $Y14_SARS2	ORF14	PF17635	bCoV_Orf14	Betacoronavirus uncharacterized protein 14 (SARS-CoV-2 like)

The Pfam HMM library can be downloaded from https://pfam.xfam.org and can be used in combination with pfam_scan to perform Pfam analysis locally. Multiple sequence alignments of matches can be generated using hmmalign (http://hmmer.org/). Precalculated matches and alignments are available from the Pfam FTP site (ftp://ftp.ebi.ac.uk/pub/databases/Pfam/releases/Pfam_SARS-CoV-2_2.0/). Pfam is freely available under the Creative Commons Zero (CC0) licence.

## Tracking, epidemiology and evolution

As there is no universal approach for classifying a virus species’ genetic diversity, the phylogenetic clades are referred to by different terms, such as ‘subtypes’, ‘genotypes’ or ‘groups’. However, phylogenetic assignment is important for studies on virus epidemiology, evolution and pathogenesis (see Covidex, Pangolin). Thus, a nomenclature system for naming the growing number of phylogenetic lineages that make up the population diversity of SARS-CoV-2 is needed. [80] have described a lineage nomenclature for SARS-CoV-2 that arises from a set of fundamental evolutionary, phylogenetic and epidemiological principles.

Phylodynamic models may aid in dating the origins of pandemics, provide insights into epidemiological parameters, e.g. $R_0$ [110], or help determine the effectiveness of virus control efforts (see BEAST 2, phylogeographic reconstruction). Phylodynamic analyses aim to conclude epidemiological processes from viral phylogenies, at the most basic level by comparing genetic relatedness to geographic relatedness.

Mathematical epidemiological models project the progress of the pandemic to show the likely outcome and help inform public health interventions (see COPASI, COVIDSIM). Such models help with analysing the effects of contact reduction measures or other interventions, forecasting hospital resource usage and guiding political decision-making.

As the pandemic progresses, SARS-CoV-2 is naturally accumulating mutations. On average, the observed changes would be expected to have no or minimal consequence for virus biology. However, tracking these changes (see CoV-GLUE, PoSeiDon) will help us better understand the pandemic and could help improve the effectiveness of antiviral drugs and vaccines, both pharmaceutical prevention measures that will be crucial to control the COVID-19 pandemic [38, 101].

### Covidex: alignment-free subtyping using machine learning

**Figure 6:**
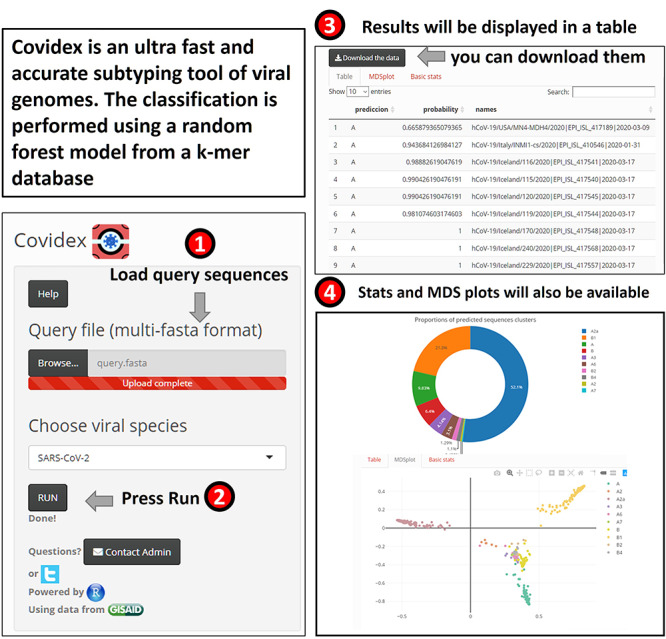
**Overview of Covidex for viral subtyping analysis.** Left: The user is expected to load a sequence file and to select the model that will be applied for classification. Models may be selected from the default list or uploaded by the user. Right: The program output (table and plots).

Viral subtypes or clades represent clusters among isolates from the global population of a defined species. Subtypification is relevant for studies on virus epidemiology, evolution and pathogenesis. Most subtype classification methods require the alignment of the input data against a set of pre-defined subtype reference sequences. These methods can be computationally expensive, particularly for long sequences such as SARS-CoV-2 ($\approx $30 kb per genome). To tackle this problem, machine learning tools may be used for virus subtyping [92]. Covidex was developed as an open-source alignment-free machine learning subtyping tool. It is a shiny app [10] that allows fast and accurate (out-of-bag error rate < 1.5 %) classification of viral genomes in pre-defined clusters (see Figure 6). For SARS-CoV-2, the default uploaded model is based on Nextstrain [31] and GISAID data [18]. Alternatively, user-uploaded models can be used. Covidex is based on a fast implementation of random forest trained over a k-mer database [7, 118]. By training the classification algorithms over k-mer frequency vectors, Covidex substantially reduces computational and time requirements and can classify hundreds of SARS-CoV-2 genomes in seconds. Thus, in the context of the current global pandemic where the number of available SARS-CoV-2 genomes is growing exponentially, SARS-CoV-2 research can benefit from this specific tool designed to reduce the time needed in data analysis significantly.

Covidex is available via SourceForge under GPLv3: https://sourceforge.net/projects/covidex or the web application https://cacciabue.shinyapps.io/shiny2/.

### Pangolin: Phylogenetic Assignment of Named Global Outbreak LINeages

Pangolin assigns a global lineage to query SARS-CoV-2 genomes by estimating the most likely placement within a phylogenetic tree of representative sequences from all currently defined global SARS-CoV-2 lineages based on the lineage nomenclature proposed by [80]. It is easily scalable so that it can be run on either thousands or a handful of sequences. Internally, pangolin runs mafft [42] and iqtree [67, 68], providing a guide tree and alignment to keep analysis overhead relatively lightweight.

Pangolin has many applications, including frontline hospital use and local and global surveillance. For example, in hospitals sequencing SARS-CoV-2 samples, it could be used to rule out within-hospital transmission, informing infection control measures. It can also be used for surveillance purposes, summarizing which lineages are present in an area of interest. The web-application also connects with Microreact (microreact.org) displaying query sequences in the context of the global lineages worldwide. pangolin is used as part of COG-UK’s (https://www.cogconsortium.uk/) data processing pipeline to assign lineages to UK sequences. Further, users can define their own finer-scale lineages, for instance within-country lineages, and provide their own guide tree and alignment.

Pangolin makes it easy to get useful information out of viral genome sequencing in real-time and can assist in identifying new introductions and in tracking the spread of SARS-CoV-2.

Pangolin is available via GitHub under GPLv3: https://github.com/hCoV-2019/pangolin/ and as web application via https://pangolin.cog-uk.io/.

### BEAST 2: phylodynamics based on Bayesian inference

Important evolutionary and epidemiological questions regarding SARS-CoV-2 can be addressed using Bayesian phylodynamic inference [27], which allows the adequate combination of evidence from multiple independent sources of data, such as genome sequences, sampling dates and geographic locations. BEAST 2 [6] is an advanced computational software framework that enables sophisticated Bayesian analyses utilizing a range of phylodynamic packages, e.g. [14, 45, 50, 93, 107, 108, 111]. The phylogenetic history (the tree) can be inferred simultaneously with evolutionary and epidemiological parameters, such that the uncertainty from all aspects of the joined model is accounted for and reflected in the results. Phylodynamic analysis of SARS-CoV-2 is crucial in understanding (i) SARS-CoV-2 evolutionary dynamics, particularly through estimation of the evolutionary rate at which mutations get fixated in the viral genome, (ii) the temporal origin of a selection of COVID-19 cases as an approximation of the time at which a sub-epidemic emerged, (iii) the geographical origin of sub-epidemics, (iv) SARS-CoV-2 transmission dynamics, e.g. through direct estimation of the effective reproduction number $R_e$ and its changes through time, and (v) the proportion of undetected COVID-19 cases. Indeed, due to the evolutionary and epidemiological processes occurring on the same time scale, the diversity in the viral genome sheds light on between-host transmission dynamics - making Bayesian phylodynamic analysis of SARS-CoV-2 a crucial complement to classical epidemiological methods.

BEAST 2 is available via https://www.beast2.org/ under the GNU Lesser General Public License (LGPL).

### Phylogeographic reconstruction using air transportation data

Phylogeographic methods combine genomic data with the sampling locations of viral isolates and models of spread, e.g. using air travel or local diffusion, to reconstruct the putative spread paths and outbreak origins of rapidly evolving pathogens. [82] published a method that infers locations for internal nodes of a phylogenetic tree using a parsimonious reconstruction together with effective distances, as defined by [8]. Effective distances are calculated based on passenger flows between airports. A strong connection between two airports is represented by a small distance. Using these distances as a cost matrix, the parsimonious reconstruction identifies ancestral locations for internal nodes of the tree that minimize the distances along the phylogeny. This method allows rapid inferences of spread paths on a fine-grained geographical scale [82]. Reconstruction using effective distances infers phylogeographic spread more accurately than reconstruction using geographic distances or Bayesian reconstructions that do not use any distance information.

Phylogeographic reconstruction using air transportation data can be used to study the global spread of the SARS-CoV-2 pandemic, especially in the early phases when air travel still substantially contributed to the spread of the virus. The method is currently adapted to consider both air travel and local movement data within countries during inference to reflect the changing worldwide movements in different phases of the pandemic.

The code is included in the GitHub repository for [82] under APLv2: https://github.com/hzi-bifo/Phylogeography_Paper

### COPASI: modeling SARS-CoV-2 dynamics with differential equations

COPASI is a dynamics simulator, originally focused on chemical and biochemical reaction networks [37]. However, it is by now also widely applied to other fields, including epidemiology. It allows simulating models with the traditional differential equation approach that represents populations as continua, as well as with a stochastic kinetics approach which considers populations are composed of individuals. COPASI has a common model representation for both these approaches, which allows switching between them with ease. Additionally, one can add arbitrary discrete events to models. This software is equipped with several algorithms that provide comprehensive analyses of models, and it has support for parameter estimation using a series of optimization algorithms. COPASI has been used to model various aspects of virology, including mechanisms of action [22, 88, 97, 103], pharmaceutical interventions [1], virus life-cycle [4], vaccine design [39] and dynamics of epidemics [2, 124, 125]. COPASI has also been applied to COVID-19, particularly to model the dynamics of the epidemic and effect of interventions [116]. Some of the authors have also used COPASI to model the local epidemics and forecast usage of hospital resources (P. Mendes) and to compare the possible advantages of contact network agent-based models over differential equation models (S. Hoops).

COPASI is available from http://copasi.org/ and https://github.com/copasi under the Artistic License 2.0.

### COVIDSIM: epidemiological models of viral spread

**Figure 7:**
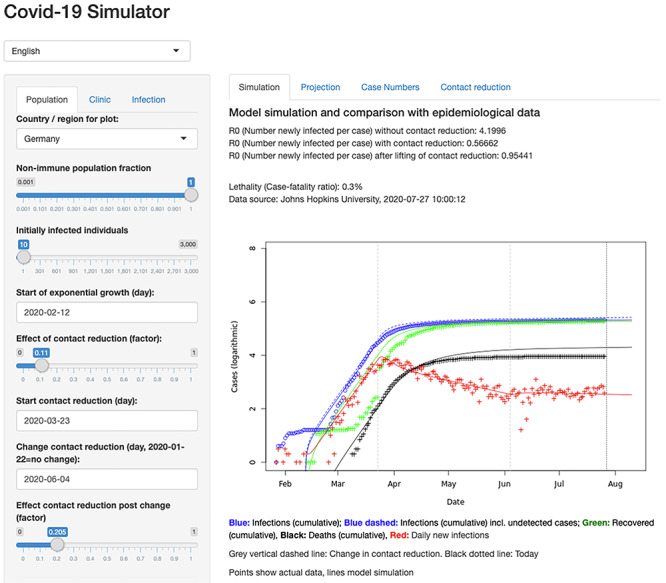
**Web interface of the COVIDSIM simulator**. The interface is allowing the user to modify model parameters and compare simulated dynamics with real infection data.

Classical epidemiological models have seen broad reuse in describing the COVID-19 outbreak. Deterministic or compartmental mathematical models assign individuals in a population to different subgroups and describe their dynamic changes using systems of differential equations. For SARS-CoV-2, the SEIR model and extended versions thereof are frequently used. The underlying model framework is not new at all, and related models have been described already at the beginning of the 20^th^ century to model infectious diseases [43]. In brief, in the SEIR or SEIRD-Model, individuals in a population are grouped into *Susceptible* (S), *Exposed* (E), *Infected* (I), *Recovered* (R) and *Deceased* (D) individuals. Initially, all individuals except for a small number who are already infected are considered susceptible to infection. The model can then simulate the population infection dynamics, using parameters such as the incubation time or the average disease duration for parameterization of the differential equations. Such SEIR models have been used to predict the COVID-19 dynamics, e.g. in Spain and Italy, and to analyse the effect of control strategies [60]. Extended versions of the SEIR model were developed to guide political decision-making [44]. For example, in Germany, this model is implemented in COVIDSIM, including hospitalized patients and patients in intensive care and implementing effects of contact reduction measures. It can be overlaid with data from different German federal states and data from other countries. This model has a convenient web interface (see Figure 7), permitting the user to change model parameters and get an intuitive feeling for the model dynamics – allowing it to estimate infection parameters and to analyse effects of contact reduction measures and guide political decision-making.

The web interface is available via http://www.kaderali.org:3838/covidsim.

### CoV-GLUE: tracking nucleotide changes in the SARS-CoV-2 genome

**Figure 8:**
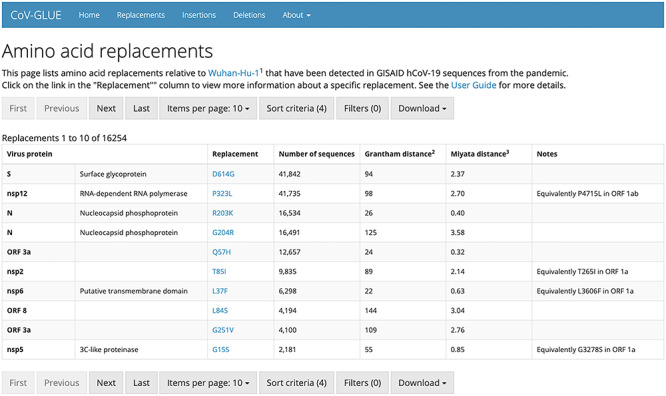
**List of amino acid replacements to the SARS-CoV-2 reference sequence.** Replacements have been detected in GISAID SARS-CoV-2 sequences from the pandemic using CoV-GLUE.

SARS-CoV-2 is naturally accumulating nucleotide mutations in its RNA genome as the pandemic progresses. Point mutations, specifically non-synonymous substitutions, will result in amino acid replacements in viral genome sequences, while other mutations will result in insertions or deletions (indels). On average the observed changes would be expected to have no or minimal consequence for virus biology. However tracking these changes will help us better understand and control the pandemic as mutations could arise with impact on virus biology and could lead to escape from antiviral drugs and future vaccines. The purpose of CoV-GLUE is to track the changes accumulating in the SARS-CoV-2 genome (see Figure 8). The resource was developed exploiting GLUE, a data-centric bioinformatics environment for virus sequence data, with a focus on variation, evolution and sequence interpretation [90]. Sequences are downloaded from GISAID EpiCoV [89] approximately every week and added to a constrained alignment within the GLUE framework. Users can browse the accumulating variation or submit a FASTA file of a novel genome to CoV-GLUE for comparison to the available data. An amino acid replacements, indels and diagnostic primer design report is generated from the submitted data. The user can access the detected variants and using a phylogenetic placement maximum-likelihood method [94] visualize their sequence relative to a reference data set. The user’s sequence is also assigned to a lineage consistent with [80].

CoV-GLUE will help advance SARS-CoV-2 research by tracking changes accumulating in the SARS-CoV-2 genome. CoV-GLUE web application is available online via http://cov-glue.cvr.gla.ac.uk/. CoV-GLUE is not released as an open source installable GLUE package due to the legal restrictions on GISAID data (see Section Detection and annotation). The underlying software system GLUE is open source and licensed under the GNU Affero General Public License v3.0 (AGPLv3).

### PoSeiDon: Positive Selection Detection and Recombination Analysis

Viruses and their hosts are in constant competition, and selection pressure continuously affects the evolution of their genes. Selection pressure, in the form of positive selection, can be studied by comparing the rates of non-synonymous (dN) and synonymous substitutions (dS) in an alignment of orthologous genes. Over several sites (codons), the dN/dS ratio can reach values well above 1 [121]. Such positively selected sites are described in recent SARS-CoV-2 studies. For example, [109] showed that the selection pressure on ORF3a and ORF8 genes can drive the evolution of the virus during the COVID-19 pandemic, while [47] describe worrying changes in the spike protein through the detection of positive selection.

PoSeiDon simplifies the detection of positive selection in protein-coding sequences [36]. Firstly, the pipeline builds a multiple sequence alignment, estimates a best-fitting substitution model and performs a recombination analysis followed by the construction of all corresponding phylogenies. Secondly, positively selected sites under varying models are detected. The results are summarized in a user-friendly web page, providing all intermediate results and graphically displaying recombination events and positively selected sites.

The rapid detection of positive selection helps to monitor protein changes of SARS-CoV-2 during the pandemic. It provides potential target sites for drug development, helping to counteract the virus during its ”arms race” with the human species.

Poseidon is available via GitHub under MIT License: https://github.com/hoelzer/poseidon.

## Drug design

To limit the pandemic threat, it is of utmost importance to develop therapy and vaccination strategies against COVID-19. Understanding the molecular mechanisms underlying the disease’s pathogenesis is key to identifying potential drug candidates for clinical trials. Viral-host protein-protein interactions (PPIs) play a crucial role during viral infection and hold promising therapeutic prospects.

To facilitate the identification of potential drugs, a screening of known drugs and PPIs, referred to as drug repurposing, is usually cheaper and more time-efficient than designing drugs from scratch [41, 86]. This is especially true for SARS-CoV-2, as it is a member of a viral genus that has been thoroughly studied. Therefore, we can infer information and potential drug targets from other *betacoronaviruses*, and especially SARS-CoV-1. The described databases contain information about virus-host PPIs (see VirHostNet, CoVex) and virus-drug interactions (see CORDITE, CoVex) and gather information from other viruses and drugs to infer potential PPIs for SARS-CoV-2 (see CoVex, P-HIPSTer).

### VirHostNet SARS-CoV-2 release

The complete understanding of molecular interactions between SARS-CoV-2 and host cellular proteins is key to highlight functions that are essential for viral replication and pathogenesis of COVID-19 outbreak. Toward this end, VirHostNet [30] was upgraded in March 2020 to include a comprehensive collection of protein-protein interactions manually annotated from the literature involving ORFeomes from multiple coronaviruses, including MERS-CoV, SARS-CoV-1 and SARS-CoV-2. This biocuration effort also incorporated, in close to real-time, the data obtained through affinity-purification mass spectrometry by the Korgan laboratory [26]. Hence, in a few days, more than 650 binary protein-protein interactions were made available to scientists working on COVID-19.

The VirHostNet resource was rapidly catalogued as a fair and open data resource to help fight against COVID-19 [84]. To leverage the cost of highly expensive experiments, open access is provided to the interology web application allowing fast and reproducible *in silico* prediction of SARS-CoV-2/human interactome The interactome predicted for SARS-CoV-2 was wired to an anti-apoptotic switch regulated by Bcl-2 family members that could potentially be a therapeutic target. The network reconstruction identified the prosurvival protein Bcl-xL and the autophagy effector Beclin 1 as vulnerable nodes in the host cellular defense system against SARS-CoV-2. Interestingly, both proteins harbour a so-called Bcl-2 homology 3 (BH3)-like motif, which is involved in homotypic (inside the Bcl-2 family) and heterotypic interactions with other domains.

The VirHostNet SARS-CoV-2 release will accelerate research on the molecular mechanisms underlying virus replication as well as COVID-19 pathogenesis and will provide a systems virology framework for prioritizing drug candidates repurposing.

VirHostNet web application is available via http://virhostnet.prabi.fr/. All data is open access.

### CORDITE: CORona Drug InTERactions database

CORDITE collects data on potential drugs, targets and their interactions for SARS-CoV-2 from published articles and preprints [62]. CORDITE integrates many functionalities to enable users to access, sort and download relevant data to conduct meta-analyses, to design new clinical trials or even to conduct a curated literature search. CORDITE automatically incorporates publications from PubMed (https://www.ncbi.nlm.nih.gov/pubmed/), bioRxiv (https://www.biorxiv.org/), chemRxiv https://www.chemrxiv.org/) and medRxiv https://www.medrxiv.org/) that report information on computational, *in vitro*, or case studies on potential drugs for COVID-19. Besides original research, reviews and comments are also included in the database. The information from the articles and preprints are manually curated by moderators and can be accessed via the web server or the open API. Moreover, registered clinical trials from the NIH https://clinicaltrials.gov/) for COVID-19 are also included. Users can directly access the publications, interactions, drugs, targets and clinical trials, and thus the data can be easily integrated into other software or apps.

The CORDITE database is updated weekly and, at the date of submission, provides data for more than 700 interactions of 23 targets for more than 530 drugs from almost 300 publications and more than 240 clinical trials (as of May 19, 2020). It is thus the largest, curated database available for drug interactions for SARS-CoV-2. It allows researchers to carry out meta-analyses on potential drugs systematically and to identify potential drug candidates for clinical trials.

CORDITE can be accessed via https://cordite.mathematik.uni-marburg.de (CC BY-ND).

### CoVex: CoronaVirus Explorer

**Figure 9:**
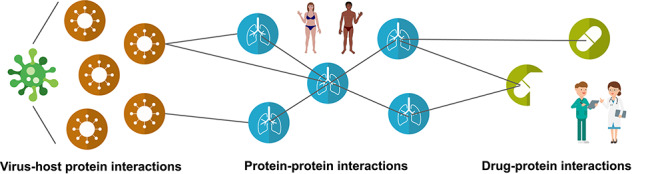
**CoVex: CoronaVirus Explorer.**CoVex is a network medicine web platform that allows its users to interactively mine a large interactome that integrates information about virus–host protein interactions, known human protein–protein interactions as well as drug–protein interactions. CoVex can be used for identifying potential drug targets and drug repurposing candidates.

CoVex [83] is a network and systems medicine web platform that integrates experimental virus-human protein interactions for SARS-CoV-2 [26] and SARS-CoV-1 [30, 75], human protein-protein interactions [49] and drug-protein interactions [24, 65, 106, 114, 117, 122] into a large-scale interactome (see Figure 9). It allows biomedical and clinical researchers to predict novel drug targets as well as drug repurposing candidates using several state-of-the-art graph analysis methods specifically tailored to the network medicine context. Here, expert knowledge about virus replication, immune-related biological processes or drug mechanisms can be applied to compile a set of host or viral proteins (referred to as seeds). Alternatively, users can upload a list of proteins (e.g. differentially expressed genes, a list of proteins related to a molecular mechanism of interest) or proteins targeted by drugs of interest (e.g. a set of drugs known to be effective) as seeds to guide the analysis. Based on the selected seeds, CoVex offers three main actions: (1) searching the human interactome for viable drug targets, (2) identifying repurposable drug candidates and (3) a combination of actions, i.e. starting from a selection of virus or virus-interacting proteins, users can mine the interactome for suitable drug targets for which, in turn, suitable drugs are identified. In summary, CoVex allows researchers to systematically identify already approved drugs that could be repurposed to treat SARS-CoV-2, which is faster than developing new drugs from scratch.

CoVex web application is available via https://exbio.wzw.tum.de/covex/.

### P-HIPSTer: a virus–host protein–protein interaction resource

Viral-host protein-protein interactions (PPIs) play a crucial role during viral infection by co-opting host cellular processes and hold promising therapeutic prospects. Along these lines, the P-HIPSTer database can significantly contribute to SARS-CoV2 research by providing: (1) testable hypotheses on molecular interactions underlying viral infection and pathogenesis and (2) highlighting host factors and pathways that serve as potential drug targets to treat infection caused by different coronaviruses.

**Figure 10:**
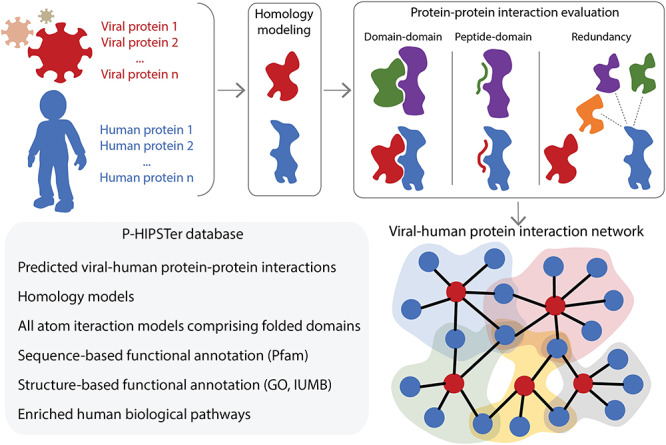
P-HIPSTer combines sequence and structural information to predict viral-host PPIs. P-HIPSTer evaluates the likelihood ratio (LR) for the potential interaction between a viral protein (in red) and a human protein (in blue) combining three evidences: (i) domain–domain LR that two structure domains interact based on known complex (green and purple domain–domain complex) comprised of their structural neighbours; (ii) peptide–domain LR that an unstructured peptide in one query binds to a structured domain in the second query based on known binding motifs/peptide–domain complex (green and purple peptide–domain complex) using both sequence and structural similarity; (iii) redundancy LR based on evidence that multiple structural neighbours (in orange, purple and green) of one query protein is known to interact with the remaining query protein. Each viral protein is functionally annotated based on sequence and structural similarity (either using homology models or known protein structures) and their corresponding set of predicted interacting human proteins.

P-HIPSTer comprises $\sim $282,000 predicted viral-human PPIs on $\sim $1,000 viruses with an experimental validation rate of $\sim $76% [52]. Its predictive algorithm is an adaptation of PrePPI [21, 123] and combines sequence and structural information to infer viral-human PPIs mediated by domain-domain or peptide-domain contacts (see Figure 10). In addition, P-HIPSTer builds all-atom interaction models for high-confidence PPI predictions involving folded domains and integrates sequence- and structure-based functional annotations for viral proteins at multiple levels, including host biological pathways based on the predicted PPIs [3, 20, 23, 96]. Hence, P-HIPSTer constitutes a complimentary resource to high-throughput experimental approaches [26]. As of April 2020, P-HIPSTer contains predictions for 15 coronaviruses with varying pathogenic potential (alpha- and betacoronaviruses) and reports 4,587 viral-host PPIs involving 397 human proteins. This unique collection of predicted viral-human PPIs enables the discovery of PPIs commonly employed within the *Coronaviridae* family and PPIs associated with their pathogenicity.

The database is available via http://www.phipster.org/

## Concluding remarks

Bioinformaticians around the world have reacted quickly to the COVID-19 pandemic by providing coronavirus-specific tools to advance SARS-CoV-2 research and boost the detection, understanding and treatment of COVID-19. This review does not claim to be complete, and in light of the rapid ongoing research, further tools will be developed.

Efficient response to the pandemic requires high-quality SARS-CoV-2 data and meta-data [87] and newly released software to be available freely and as open source. Open source code invites other developers to improve the software. Preferably, code should be shared via a suitable repository such as GitHub, allowing for transparency and managing versioning and feature development. In particular, when software and resources are evolving as fast as the virus, versioning and reproducibility of all steps are of increasing importance. In the context of pipelines, versions of third-party tools should be fixed using package managers like Conda or by encapsulation using container software (Docker [5], Singularity). Workflow management systems such as SnakeMake or Nextflow [19, 100] allow easy installation and reproducible execution on various platforms. In the best case, all tools and pipelines should be automatically and continuously tested to evaluate their quality and usability. Also, manuscripts on software and methods development can be made available as preprints to accelerate their dissemination. Of course, these standards are not specific for coronavirus related research, but rather general points about bioinformatics software.

One major bottleneck hindering high software standards is the limited capacity of scientists to build versatile software, rather than prototypes. This might be improved by merging projects with similar or overlapping goals. However, this requires a central overview of newly developed tools and ongoing research projects and of how (future) products may fit together, e.g. in the form of a processing pipeline. Unfortunately, the life cycle of software in research is relatively short. Usually, scientific funding does not include the continuous maintenance of tools and pipelines so that developers are forced to move on to other projects and research grants.

The European Virus Bioinformatics Center curates a list of bioinformatics tools specifically for coronaviruses (http://evbc.uni-jena.de/tools/coronavirus-tools/), some of which were presented in this review. Other initiatives are collecting relevant datasets (COVID-19 Data Portal, https://www.covid19dataportal.org/) or are supporting researchers by offering assistance with SARS-CoV-2 genome sequencing (NFDI4Microbiota, https://nfdi4microbiota.de/index.php/covid-19/). ELIXIR (https://elixir-europe.org/services/covid-19) provides a range of services to study SARS-CoV-2, in particular, the European Galaxy server for data-intensive research that provides access to scientific tools and training materials to guide users through COVID-19 data analysis. In addition, it is an encouraging development seeing researchers joining efforts in national and international initiatives to combat the ongoing pandemic. For example, researchers around the world are jointly reconstructing the molecular processes of the virus-host interactions to develop a COVID-19 Disease Map [74].

**Key Points**

- In light of the sheer amount of data, many fundamental questions in SARS-CoV-2 research can only be tackled with the help of bioinformatic tools.
- Bioinformatic analysis of SARS-CoV-2 data has the potential to track and trace SARS-CoV-2 sequence evolution and identify potential drug targets.
- All tools are free to use and available online to rapidly advance SARS-CoV-2 research.

## Supplementary Material

nawrocki-nih-coversheet_bbaa232

main_tracked_changes_bbaa232

## Availability

All presented tools are free to use and available online, either through web applications or public code repositories. Licenses are given in Table 1. You can find a list of the presented tools and further tools on the EVBC website: http://evbc.uni-jena.de/tools/coronavirus-tools/

## Notes

**Franziska Hufsky** is a postdoctoral researcher at Friedrich-Schiller-University Jena, Germany. She is coordinating the European Virus Bioinformatics Center.

**Kevin Lamkiewicz** is a PhD student at Friedrich-Schiller-University Jena, Germany. His research focuses on viral RNA secondary structures and their role in the life-cycle of viruses.

**Alexandre Almeida** is a Postdoctoral Fellow at the EMBL-EBI and the Wellcome Sanger Institute, UK, investigating the diversity of the human gut microbiome using metagenomic approaches.

**Abdel Aouacheria** is researcher at CNRS, France. He has been working for more than twenty years on cell suicide (apoptosis) with a growing interest in transdisciplinary research approaches (e.g. biochemistry, cell biology, evolution, epistemology).

**Cecilia Arighi** is the Team Leader of Biocuration and Literature Access at PIR, USA. Her responsibilities include improving coverage and access to literature and annotations in UniProt via text mining, integration from external sources and community crowdsourcing.

**Alex Bateman** is the Head of Protein Sequence Resources at EMBL-EBI, UK, where he is responsible for numerous protein and non-coding RNA sequence and family databases.

**Jan Baumbach** is Chair of Experimental Bioinformatics and Professor at Technical University of Munich, Germany. His research is focused on Network and System Medicine as well as privacy-aware artificial intelligence in health and medicine.

**Niko Beerenwinkel** is Professor of Computational Biology at ETH Zurich, Switzerland. His research is focused on developing statistical and evolutionary models for high-throughput molecular profiling data in oncology and virology.

**Christian Brandt** is a postdoc at the Institute of Infectious Disease and Infection Control at Jena University Hospital, Germany. His research focuses on nanopore sequencing and the development of complex workflows to answer clinical questions in the field of metagenomics, bacterial infections, transmission, spread, and antibiotic resistance.

**Marco Cacciabue** is a postdoctoral fellow of the Consejo Nacional de Investigaciones Científicas y Tócnicas (CONICET) working on FMDV virology at the Instituto de Agrobiotecnología y Biología Molecular (IABiMo, INTA-CONICET) and at the Departamento de Ciencias Básicas, Universidad Nacional de Luján (UNLu), Argentina.

**Sara Chuguransky** is Biocurator for Pfam and InterPro databases, at the EMBL-EBI, UK.

**Oliver Drechsel** is a permanent researcher in the core facility of the bioinformatics department at the Robert Koch-Institute, Germany.

**Robert D. Finn** leads EMBL-EBI’s Sequence Families team, which is responsible for a range of informatics resources, including Pfam and MGnify. His research is focused on the analysis of metagenomes and metatranscriptomes, especially the recovery of genomes.

**Adrian Fritz** is a doctoral researcher in the Computational Biology of Infection Research group of Alice C. McHardy at the Helmholtz Centre for Infection Research, Germany. He mainly studies metagenomics with a special focus on strain-aware assembly.

**Stephan Fuchs** is coordinator of the core facility of the bioinformatics department at the Robert Koch-Institute, Germany.

**Georges Hattab** heads the group Data Analysis and Visualization and the Bioinformatics Division at Philipps-University Marburg, Germany. His research is focused on information related tasks: theory, embedding, compression, and visualization.

**Anne-Christin Hauschild** is a postdoctoral researcher at Philipps-University Marburg, Germany. Her research focuses on federated machine learning.

**Dominik Heider** is Professor for Data Science in Biomedicine at the Philipps-University of Marburg, Germany at the Faculty of Mathematics and Computer Science. His research is focused on machine learning and data science in biomedicine, in particular for pathogen resistance modeling.

**Marie Hoffmann** is a PhD student at Freie Universität Berlin, Germany in the Department of Mathematics and Computer Science and expects to complete by 2020. Her current research centers around the implementation of bioinformatical methods to build tools that enable planning and evaluation of metagenomic experiments.

**Martin Hölzer** is a post-doctoral researcher and team leader at the Friedrich Schiller University Jena, Germany. His research is focused on the detection of viruses from DNA and RNA sequencing data (the longer the better).

**Stefan Hoops** is a research associate professor at the Biocomplexity Institute and Initiative at the University of Virginia, USA. His research focus is simulation (Epidemiology, Immunology), software tools (COPASI) and standards (SBML).

**Lars Kaderali** is full Professor for Bioinformatics and head of the Institute of Bioinformatics at University Medicine Greifswald, Germany. His research focus is on mathematical modelling of molecular and cellular processes, with a special focus on modeling viral infection.

**Ioanna Kalvari** is a Senior Software Developer at EMBL-EBI responsible for the Rfam database.

**Max von Kleist** is the head of the bioinformatics department at the Robert Koch-Institute, Germany.

**Renó Kmiecinski** is an assistant in the core facility of the bioinformatics department at the Robert Koch-Institute, Germany.

**Denise Kühnert** leads an independent research group at the Max Planck Institute for the Science of Human History. Her scientific focus is in the area of phylodynamics, where she aims for a broader understanding of infectious disease dynamics of modern and ancient pathogen outbreaks.

**Gorka Lasso** is a Research Assistant Professor at the Chandran Lab, Albert Einstein College of Medicine, USA. His research is focused on modeling viral-host protein-protein interactions.

**Pieter Libin** is a postdoctoral researcher at the Data Science institute of the University of Hasselt, Belgium. His research is focused on investigating prevention strategies to mitigate viral infectious diseases.

**Markus List** heads the group of Big Data in Biomedicine at the Technical University of Munich, Germany. His group combines systems biomedicine and machine learning to integrate heterogeneous omics data.

**Hannah F. Löchel** is a PhD student at Philipps-University Marburg, Germany. Her research focuses on machine learning methods for pathogen resistance prediction.

**Maria J. Martin** is the Team Leader of Protein Function development at EMBL-EBI, UK, where she leads the bioinformatics and software development of UniProt. Her research focuses on computational methods for protein annotation.

**Roman Martin** is a PhD student at Philipps-University Marburg, Germany. His research focuses on bioinformatics pipelines for genome assembly.

**Julian Matschinske** is a PhD candidate at the Chair of Experimental Bioinformatics at TU Munich, Germany. His research is mainly focused on federated machine learning and data privacy in conjunction with federated systems.

**Alice C. McHardy** leads the Computational Biology of Infection Research Lab at the Helmholtz Centre for Infection Research in Braunschweig, Germany. She studies the human microbiome, viral and bacterial pathogens, and human cell lineages within individual patients by analysis of large-scale biological and epidemiological data sets with computational techniques.

**Pedro Mendes** is a Professor of Cell Biology at the Center for Quantitative Medicine of the University of Connecticut School of Medicine, USA. His research is focused on computational systems biology.

**Jaina Mistry** is a developer for the Pfam database at EMBL-EBI, UK. She runs the production pipeline for Pfam.

**Vincent Navratil** is a technical leader in Bioinformatics and Systems Biology at the Rhône Alpes Bioinformatics core facility, Universitó de Lyon, France. His research focuses on virus/host systems biology and NGS data analysis.

**Eric P. Nawrocki** is a staff scientist at the National Center for Biotechnology Information (NCBI). He is part of the Rfam team and lead developer of the Infernal software package for RNA sequence analysis and VADR for viral sequence annotation.

**Áine Niamh O’Toole** is a PhD student in the Rambaut group at Edinburgh University, UK. As part of the ARTIC Network, her research is focused on virus evolution and real-time molecular epidemiology of viral outbreaks.

**Nancy Ontiveros-Palacios** is biocurator for the Rfam database at the EMBL-EBI, UK.

**Anton I. Petrov** is the RNA Resources Project Leader at EMBL-EBI, UK. He coordinates the development of the Rfam and RNAcentral databases for non-coding RNA.

**Guillermo Rangel-Pineros** is a postdoc at the GLOBE Institute in the University of Copenhagen, Denmark. His research is focused on the development of computational pipelines for the discovery and characterization of novel bacteriophages.

**Nicole Redaschi** is the head of Development of the Swiss-Prot group at the SIB for UniProt and SIB resources that cover viral biology (ViralZone), enzymes and biochemical reactions (ENZYME, Rhea) and protein classification/annotation (PROSITE, HAMAP).

**Susanne Reimering** is a doctoral researcher in the Computational Biology of Infection Research group of Alice C. McHardy at the Helmholtz Centre for Infection Research. She studies viral phylogenetics, evolution and phylogeography with a focus on influenza A viruses.

**Knut Reinert** is a professor for algorithmic bioinformatics at Freie Universität Berlin, Germany. His research aims at enabling translational research by removing existing (communication) gaps between theoretical algorithmicists, statisticians, programmers and users in the biomedical field.

**Alejandro Reyes** is an associate professor at Universidad de los Andes, Colombia, where he leads the Computational Biology and Microbial Ecology Research Group focusing on viruses and microbial metagenomic and computational research.

**Lorna Richardson** is the content coordinator for the Sequence Families team at EMBL-EBI, UK, covering a range of resources including Pfam.

**David L. Robertson’s** research interests focus on computational and data-driven approaches applied to viruses and their host interactions. He has over 25 years of experience of studying molecular evolution and is currently head of the bioinformatics group at the MRC-University of Glasgow Centre for Virus Research, UK.

**Sepideh Sadegh** is a PhD student in the Chair of Experimental Bioinformatics at Technical University of Munich, Germany. Her research area is focused on Network medicine, more specifically network-based drug repurposing

**Joshua B. Singer** is a Research Software Engineer at the MRC-University of Glasgow Centre for Virus Research, Glasgow, Scotland, UK. He is the lead developer of the GLUE software system for virus genome sequence data analysis.

**Kristof Theys** is a senior researcher at the Rega institute of the University of Leuven, Belgium. His work is oriented towards clinical and epidemiological virology, with an emphasis on studies of within-host evolutionary and between-host transmission dynamics.

**Chris Upton** is a professor in the Department of Biochemistry and Microbiology, University of Victoria, Canada, focusing on the comparative genomics of large viruses and development of bioinformatics tools for their analysis.

**Marius Welzel** is a PhD student at Philipps-University Marburg, Germany. His research focuses on codes for DNA storage systems.

**Lowri Williams** is a biocurator for the Pfam and InterPro databases, at EMBL-EBI, UK.

**Manja Marz** is a professor for RNA bioinformatics at Friedrich Schiller University Jena, Germany, and managing director of the European Virus Bioinformatics Center. Her research focusses on RNA bioinformatics, high-throughput analysis and virus bioinformatics.
