# Supplementary material for: Computational strategies to combat COVID-19: useful tools to accelerate SARS-CoV-2 and coronavirus research
Source: Brief Bioinform. 2020 Nov 4;22(2):642–63. doi: 10.1093/bib/bbaa232 (PMC7665365; doi:10.1093/bib/bbaa232)
Supplement: main_tracked_changes_bbaa232 [file main_tracked_changes_bbaa232.pdf]

Computational strategies to combat COVID-19: Useful tools to accelerate SARS-CoV-2 and Coronavirus research

Franziska Hufsky [1,2,*], Kevin Lamkiewicz [1,2,*], Alexandre Almeida [3,4], Abdel Aouacheria [5], Cecilia Arighi [6], Alex Bateman [3], Jan Baumbach [7], Niko Beerenwinkel [8,9,2], Christian Brandt [10], Marco Cacciabue [11,12], Sara Chuguransky [3], Oliver Drechsel [13], Robert D. Finn [3], Adrian Fritz [14], Stephan Fuchs [13], Georges Hattab [15], Anne-Christin Hauschild [15], Dominik Heider [15,2], Marie Hoffmann [16], Martin Hölzer [1,2], Stefan Hoops [17], Lars Kaderali [18,2], Ioanna Kalvari [3], Max von Kleist [13,2], René Kmiecinski [13], Denise Kühnert [19,2], Gorka Lasso [20], Pieter Libin [21,22,23], Markus List [7], Hannah F. Löchel [15], Maria J. Martin [3], Roman Martin [15], Julian Matschinske [7], Alice C. McHardy [14,24,2], Pedro Mendes [25], Jaina Mistry [3], Vincent Navratil [26,2], Eric P. Nawrocki [27], Áine Niamh O'Toole [28], Nancy Ontiveros-Palacios~~Palacios-Ontiveros~~ [3], Anton I. Petrov [3], Guillermo Rangel-Pineros [29,30], Nicole Redaschi [31], Susanne Reimering [14], Knut Reinert [16,2], Alejandro Reyes [30], Lorna Richardson [3], David L. Robertson [32,2], Sepideh Sadegh [7], Joshua B. Singer [32], Kristof Theys [23,2], Chris Upton [33,2], Marius Welzel [15], Lowri Williams [3], and Manja Marz [1,2,*]

* *To whom correspondence should be addressed.*

[1] RNA Bioinformatics and High-Throughput Analysis, Friedrich Schiller University Jena, Jena, 07743, Germany
[2] European Virus Bioinformatics Center, Friedrich Schiller University Jena, Jena, 07743, Germany
[3] European Molecular Biology Laboratory, European Bioinformatics Institute (EMBL-EBI), Wellcome Genome Campus, Hinxton, UK.
[4] Wellcome Sanger Institute, Wellcome Genome Campus, Hinxton, UK.
[5] ISEM, Institut des Sciences de l'Evolution de Montpellier, Université de Montpellier, CNRS UMR 5554, Montpellier, 34095, France
[6] Protein Information Resource, University of Delaware, Newark, DE 19711, USA
[7] Chair of Experimental Bioinformatics, TUM School of Life Sciences Weihenstephan, Technical University of Munich, Freising, 85354, Germany
[8] Department of Biosystems Science and Engineering, ETH Zurich, 4058 Basel, Switzerland
[9] SIB Swiss Institute of Bioinformatics, 4058 Basel, Switzerland
[10] Institute of Infectious Disease and Infection Control, Jena University Hospital, Jena, 07747, Germany
[11] Instituto de Agrobiotecnología y Biología Molecular, INTA-CONICET, Hurlingham, Argentina
[12] Universidad Nacional de Luján, Departamento de Ciencias Básicas, Luján, Argentina
[13] MF1 Bioinformatics, Robert Koch Institute, Berlin, 13353, Germany
[14] Department for Computational Biology of Infection Research, Helmholtz Centre for Infection Research, Braunschweig, 38124, Germany
[15] Department of Mathematics and Computer Science, Philipps-University of Marburg, Marburg, 35032, Germany
[16] Algorithmic Bioinformatics, Freie Universität Berlin, Berlin, 14195, Germany
[17] Biocomplexity Institute & Initiative, University of Virginia, Charlottesville, VA 22904-4298, USA
[18] Institute for Bioinformatics, University Medicine Greifswald, Greifswald, 17475, Germany
[19] Transmission, Infection, Diversification & Evolution Group (TIDE), Max-Planck Institute for the Science of Human History (MPI-SHH), Jena, 07745, Germany
[20] Department of Microbiology and Immunology, Albert Einstein College of Medicine, Bronx, New York, 10461, USA
[21] Interuniversity Institute of Biostatistics and statistical Bioinformatics, Data Science Institute, Hasselt University, Hasselt, Belgium
[22] Artificial Intelligence lab, Department of computer science, Vrije Universiteit Brussel, Brussels, Belgium
[23] Department of Microbiology and Immunology, Rega Institute for Medical Research, Clinical and epidemiological virology, KU Leuven - University of Leuven, Leuven, Belgium
[24] German Center for Infection Research (DZIF), Braunschweig, 38124, Germany
[25] Center for Quantitative Medicine, School of Medicine, University of Connecticut, Farmington, CT 06030-6033, USA
[26] PRABI, Rhône Alpes Bioinformatics Center, Université Claude-Bernard Lyon 1, Université de Lyon, Lyon, France
[27] National Center for Biotechnology Information, National Library of Medicine, National Institutes of Health, Bethesda, MD, 20894 USA
[28] Institute of Evolutionary Biology, University of Edinburgh, Edinburgh, EH9 3FL, UK
[29] Section for Evolutionary Genomics, The GLOBE Institute, University of Copenhagen, Øster Voldgade 5-7, 1350 Copenhagen, Denmark
[30] Max Planck Tandem Group in Computational Biology, Department of Biological Sciences, Universidad de los Andes, Bogota, Colombia
[31] Swiss-Prot Group, SIB Swiss Institute of Bioinformatics, Geneva, CH-1211, Switzerland
[32] MRC-University of Glasgow Centre for Virus Research, Glasgow, G61 1QH, UK
[33] Biochemistry and Microbiology, University of Victoria, Victoria, BC V8P 5C2, Canada

Author summaries

Franziska Hufsky is a postdoctoral researcher at Friedrich-Schiller-University Jena, Germany. She is coordinating the European Virus Bioinformatics Center.

Kevin Lamkiewicz is a PhD student at Friedrich-Schiller-University Jena, Germany. His research focuses on viral RNA secondary structures and their role in the life-cycle of viruses.

Alexandre Almeida is a Postdoctoral Fellow at the EMBL-EBI and the Wellcome Sanger Institute, UK, investigating the diversity of the human gut microbiome using metagenomic approaches.

Abdel Aouacheria is researcher at CNRS, France. He has been working for more than twenty years on cell suicide (apoptosis) with a growing interest in transdisciplinary research approaches (e.g. biochemistry, cell biology, evolution, epistemology).

Cecilia Arighi is the Team Leader of Biocuration and Literature Access at PIR, USA. Her responsibilities include improving coverage and access to literature and annotations in UniProt via text mining, integration from external sources and community crowdsourcing.

Alex Bateman is the Head of Protein Sequence Resources at EMBL-EBI, UK, where he is responsible for numerous protein and non-coding RNA sequence and family databases.

Jan Baumbach is Chair of Experimental Bioinformatics and Professor at Technical University of Munich, Germany. His research is focused on Network and System Medicine as well as privacy-aware artificial intelligence in health and medicine.

Niko Beerenwinkel is Professor of Computational Biology at ETH Zurich, Switzerland. His research is focused on developing statistical and evolutionary models for high-throughput molecular profiling data in oncology and virology.

Christian Brandt is a postdoc at the Institute of Infectious Disease and Infection Control at Jena University Hospital, Germany. His research focuses on nanopore sequencing and the development of complex workflows to answer clinical questions in the field of metagenomics, bacterial infections, transmission, spread, and antibiotic resistance.

Marco Cacciabue is a postdoctoral fellow of the Consejo Nacional de Investigaciones Científicas y Técnicas (CONICET) working on FMDV virology at the Instituto de Agrobiotecnología y Biología Molecular (IABiMo, INTA-CONICET) and at the Departamento de Ciencias Básicas, Universidad Nacional de Luján (UNLu), Argentina.

Sara Chuguransky is Biocurator for Pfam and InterPro databases, at the EMBL-EBI, UK.

Oliver Drechsel is a permanent researcher in the core facility of the bioinformatics department at the Robert Koch-Institute, Germany.

Robert D. Finn leads EMBL-EBI's Sequence Families team, which is responsible for a range of informatics resources, including Pfam and MGnify. His research is focused on the analysis of metagenomes and metatranscriptomes, especially the recovery of genomes.

Adrian Fritz is a doctoral researcher in the Computational Biology of Infection Research group of Alice C. McHardy at the Helmholtz Centre for Infection Research, Germany. He mainly studies metagenomics with a special focus on strain-aware assembly.

Stephan Fuchs is coordinator of the core facility of the bioinformatics department at the Robert Koch-Institute, Germany.

Georges Hattab heads the group Data Analysis and Visualization and the Bioinformatics Division at Philipps-University Marburg, Germany. His research is focused on information related tasks: theory, embedding, compression, and visualization.

Anne-Christin Hauschild is a postdoctoral researcher at Philipps-University Marburg, Germany. Her research focuses on federated machine learning.

Dominik Heider is Professor for Data Science in Biomedicine at the Philipps-University of Marburg, Germany at the Faculty of Mathematics and Computer Science. His research is focused on machine learning and data science in biomedicine, in particular for pathogen resistance modeling.

Marie Hoffmann is a PhD student at Freie Universität Berlin, Germany in the Department of Mathematics and Computer Science and expects to complete by 2020. Her current research centers around the implementation of bioinformatical methods to build tools that enable planning and evaluation of metagenomic experiments.

Martin Hölzer is a post-doctoral researcher and team leader at the Friedrich Schiller University Jena, Germany. His research is focused on the detection of viruses from DNA and RNA sequencing data (the longer the better).

Stefan Hoops is a research associate professor at the Biocomplexity Institute and Initiative at the University of Virginia, USA. His research focus is simulation (Epidemiology, Immunology), software tools (COPASI) and standards (SBML).

Lars Kaderali is full Professor for Bioinformatics and head of the Institute of Bioinformatics at University Medicine Greifswald, Germany. His research focus is on mathematical modelling of molecular and cellular processes, with a special focus on modeling viral infection.

Ioanna Kalvari is a Senior Software Developer at EMBL-EBI responsible for the Rfam database.

Max von Kleist is the head of the bioinformatics department at the Robert Koch-Institute, Germany.

René Kmiecinski is an assistant in the core facility of the bioinformatics department at the Robert Koch-Institute, Germany.

Denise Kühnert leads an independent research group at the Max Planck Institute for the Science of Human History. Her scientific focus is in the area of phylodynamics, where she aims for a broader understanding of infectious disease dynamics of modern and ancient pathogen outbreaks.

Gorka Lasso is a Research Assistant Professor at the Chandran Lab, Albert Einstein College of Medicine, USA. His research is focused on modeling viral-host protein-protein interactions.

Pieter Libin is a postdoctoral researcher at the Data Science institute of the University of Hasselt, Belgium. His research is focused on investigating prevention strategies to mitigate viral infectious diseases.

Markus List heads the group of Big Data in Biomedicine at the Technical University of Munich, Germany. His group combines systems biomedicine and machine learning to integrate heterogeneous omics data.

Hannah F. Löchel is a PhD student at Philipps-University Marburg, Germany. Her research focuses on machine learning methods for pathogen resistance prediction.

Maria J. Martin is the Team Leader of Protein Function development at EMBL-EBI, UK, where she leads the bioinformatics and software development of UniProt. Her research focuses on computational methods for protein annotation.

Roman Martin is a PhD student at Philipps-University Marburg, Germany. His research focuses on bioinformatics pipelines for genome assembly.

Julian Matschinske is a PhD candidate at the Chair of Experimental Bioinformatics at TU Munich, Germany. His research is mainly focused on federated machine learning and data privacy in conjunction with federated systems.

Alice C. McHardy leads the Computational Biology of Infection Research Lab at the Helmholtz Centre for Infection Research in Braunschweig, Germany. She studies the human microbiome, viral and bacterial pathogens, and human cell lineages within individual patients by analysis of large-scale biological and epidemiological data sets with computational techniques.

Pedro Mendes is a Professor of Cell Biology at the Center for Quantitative Medicine of the University of Connecticut School of Medicine, USA. His research is focused on computational systems biology.

Jaina Mistry is a developer for the Pfam database at EMBL-EBI, UK. She runs the production pipeline for Pfam.

Vincent Navratil is a technical leader in Bioinformatics and Systems Biology at the Rhône Alpes Bioinformatics core facility, Université

de Lyon, France. His research focuses on virus/host systems biology and NGS data analysis.

Eric P. Nawrocki is a staff scientist at the National Center for Biotechnology Information (NCBI). He is part of the Rfam team and lead developer of the Infernal software package for RNA sequence analysis and VADR for viral sequence annotation.

Áine Niamh O'Toole is a PhD student in the Rambaut group at Edinburgh University, UK. As part of the ARTIC Network, her research is focused on virus evolution and real-time molecular epidemiology of viral outbreaks.

Nancy Ontiveros-Palacios~~Palacios-Ontiveros~~ is Biocurator for the Rfam database at the EMBL-EBI, UK.

Anton I. Petrov is the RNA Resources Project Leader at EMBL-EBI, UK. He coordinates the development of the Rfam and RNAcentral databases for non-coding RNA.

Guillermo Rangel-Pineros is a postdoc at the GLOBE Institute in the University of Copenhagen, Denmark. His research is focused on the development of computational pipelines for the discovery and characterisation of novel bacteriophages.

Nicole Redaschi is the Head of Development of the Swiss-Prot group at the SIB for UniProt and SIB resources that cover viral biology (ViralZone), enzymes and biochemical reactions (ENZYME, Rhea) and protein classification/annotation (PROSITE, HAMAP).

Susanne Reimering is a doctoral researcher in the Computational Biology of Infection Research group of Alice C. McHardy at the Helmholtz Centre for Infection Research. She studies viral phylogenetics, evolution and phylogeography with a focus on influenza A viruses.

Knut Reinert is a professor for algorithmic bioinformatics at Freie Universität Berlin, Germany. His research aims at enabling translational research by removing existing (communication) gaps between theoretical algorithmicists, statisticians, programmers, and users in the biomedical field.

Alejandro Reyes Alejandro Reyes is Associate Professor at Universidad de los Andes, Colombia, where he leads the Computational Biology and Microbial Ecology Research Group focusing on viruses and microbial metagenomic and computational research.

Lorna Richardson Lorna Richardson is the content coordinator for the Sequence Families team at EMBL-EBI, UK, covering a range of resources including Pfam.

David L. Robertson's research interests focus on computational and data-driven approaches applied to viruses and their host interactions. He has over 25 years of experience of studying molecular evolution and is currently head of the bioinformatics group at the MRC-University of Glasgow Centre for Virus Research, UK.

Sepideh Sadegh is a PhD student in the Chair of Experimental Bioinformatics at Technical University of Munich, Germany. Her research area is focused on Network medicine, more specifically network-based drug repurposing

Joshua B. Singer is a Research Software Engineer at the MRC-University of Glasgow Centre for Virus Research, Glasgow, Scotland, UK. He is the lead developer of the GLUE software system for virus genome sequence data analysis.

Kristof Theys is a senior researcher at the Rega institute of the University of Leuven, Belgium. His work is oriented towards clinical and epidemiological virology, with an emphasis on studies of within-host evolutionary and between-host transmission dynamics.

Chris Upton is a professor in the Department of Biochemistry and Microbiology, University of Victoria, Canada, focusing on the comparative genomics of large viruses and development of bioinformatics tools for their analysis.

Marius Welzel is a PhD student at Philipps-University Marburg, Germany. His research focuses on codes for DNA storage systems.

Lowri Williams is a Biocurator for the Pfam and InterPro databases, at EMBL-EBI, UK.

Manja Marz is professor for RNA bioinformatics at Friedrich Schiller University Jena, Germany, and Managing Director of the European Virus Bioinformatics Center. Her research focusses on RNA bioinformatics, high-throughput analysis and virus bioinformatics.

Abstract

SARS-CoV-2 (severe acute respiratory syndrome coronavirus 2) is a novel virus of the family *Coronaviridae*. The virus causes the infectious disease COVID-19. The biology of coronaviruses has been studied for many years. However, bioinformatics tools designed explicitly for SARS-CoV-2 have only recently been developed as a rapid reaction to the need for fast detection, understanding, and treatment of COVID-19. To control the ongoing COVID-19 pandemic, it is of utmost importance to get insight into the evolution and pathogenesis of the virus.

In this review, we cover bioinformatics workflows and tools for the routine detection of SARS-CoV-2 infection, the reliable analysis of sequencing data, the tracking of the COVID-19 pandemic and evaluation of containment measures, the study of coronavirus evolution, the discovery of potential drug targets and development of therapeutic strategies. For each tool, we briefly describe its use case and how it advances research specifically for SARS-CoV-2. All tools are free to use and~~freely~~ available online, either through web applications or public code repositories.

Contact: manja@uni-jena.de

Keywords: virus bioinformatics, SARS-CoV-2, sequencing, epidemiology, drug design, tools

1 Introduction

On December 31, 2019, the Wuhan Municipal Health Commission reported several cases of pneumonia in Wuhan (China) to the WHO[1]. The cause of these cases was a previously unknown coronavirus, now known as severe acute respiratory syndrome coronavirus 2 (SARS-CoV-2), which can manifest itself in the disease named COVID-19. At the time of writing (July 22~~May 22~~, 2020), nearly 15~~five~~ million cases were reported worldwide, with over 600~~320~~,000 deaths[2].

The group of *Coronaviridae* includes viruses with very long RNA genomes up to 33,000 nucleotides. SARS-CoV-2 belongs to the *Sarbecovirus* subgenus (genus: *Betacoronavirus*) and has a genome of approximately 30,000 nucleotides [1]. In line with other members of *Coronaviridae*, SARS-CoV-2 has four main structural proteins: spike (S), envelope (E), membrane (M), and nucleocapsid (N). Further, several nonstructural proteins are encoded in the pp1a and pp1ab polyproteins, which are essential for viral replication [1]. SARS-CoV-2 seems to use the human receptor ACE2 as its main entry [2], which has been observed for other *Sarbecoviruses* as well [3, 4]. The binding domains for ACE2 are located on the spike proteins, which further contain a novel furin cleavage site, associated with increased pathogenicity and transmission potential [5–8].

Although SARS-CoV-2 has a lower mutation rate than most RNA viruses, mutations certainly accumulate and result in genomic diversity both between and within individual infected patients. Genetic heterogeneity enables viral adaptation to different hosts and different environments within hosts, and is often associated with disease progression, drug resistance, and treatment outcome.

In light of the COVID-19 pandemic, there has been a rapid increase in SARS-CoV-2 related research. It will be critical to get insight into the evolution and pathogenesis of the virus in order to control this pandemic. Researchers around the world are investigating SARS-CoV-2 sequence evolution on genome and protein level, are tracking the pandemic using phylodynamic and epidemiological models, and are examining potential drug targets. Laboratories are sharing SARS-CoV-2 related data with unprecedented speed. In light of this sheer amount of data, many fundamental questions in SARS-CoV-2 research can only be tackled with the help of bioinformaticians. Adequate analysis of these data has the potential to boost discovery and inform both fundamental and applied science, in addition to public health initiatives.

SARS-CoV-2 is an entirely novel pathogen, and in light of the pandemic requiring a swift response to research and public health-related questions, the natural first approach is to repurpose existing methods and resources. Simultaneously, the outbreak has had a huge impact on virus bioinformatics tools that have been developed recently and it is important to understand which tools are applicable to coronaviruses, and which have been customized to address research questions related to SARS-CoV-2.

In this review, we cover bioinformatics workflows and tools (see Table 1) starting with the routine detection of SARS-CoV-2 infection, the reliable analysis of sequencing data, the tracking of the COVID-19 pandemic, the study of coronavirus evolution, up to the detection of potential drug targets and development of therapeutic strategies. All tools have either been developed explicitly for SARS-CoV-2 research, have been extended or adapted to coronaviruses, or are of particular importance to study SARS-CoV-2 epidemiology and pathogenesis.

2 Detection and annotation

The routine detection method for SARS-CoV-2 is a real-time quantitative reverse transcriptase polymerase chain reaction (qRT-PCR). The test is based on the detection of two nucleotide sequences: the virus envelope (E) gene and the gene for the RNA-dependent RNA polymerase (RdRp) [9]. Specificity (exclusion of false positives) and sensitivity (exclusion of false negatives) are two of the most important quality criteria for the validity of diagnostic tests. To ensure unique identification of SARS-CoV-2 and avoid false-negative and false-positive detection, the computation of SARS-CoV-2-specific primers is required. A new set of primers might be required, if the specificity or sensitivity of the qRT-PCR test changes due to mutations in the SARS-CoV-2 genome or related coronavirus genomes (see `PriSeT`).

Besides qRT-PCR, genome analysis plays a crucial role in public health responses, including epidemiological efforts to track and contain the outbreak (see Section **Tracking, epidemiology and evolution**). The genome sequence of SARS-CoV-2 was rapidly determined and shared on GenBank (MN908947.3). It is annotated based on sequence similarity to other coronaviruses. Next-generation sequencing (NGS) can be used to assess the genomic diversity of the virus. Regular sequencing from clinical cases is, for example, useful to monitor for mutations that might affect the qRT-PCR test (see `CoVPipe`, `V-Pipe`). To reliably derive intra-host diversity estimates from deep sequencing data is challenging since most variants occur at low frequencies in the virus population, and amplification and sequencing errors confound their detection. Multiple related viral strains (haplotypes) are hard to resolve but may be critical for the choice of therapy (see `Haploflow`, `V-Pipe`).

The SARS-CoV-2 nanopore sequencing protocol has been developed and optimised by the ARTIC network [10], which has extensive experience and ex-

[1] https://www.who.int/csr/don/05-january-2020-pneumonia-of-unkown-cause-china/en/

[2] https://www.who.int/docs/default-source/coronaviruse/situation-reports/20200722-covid-19-sitrep-184.pdf

Table 1: Bioinformatics tools accelerating SARS-CoV-2 research. Overview of all workflows and tools covered in this review. All tools are free to use and available online. A list of these and further tools can be found on the website of the European Virus Bioinformatics Center (EVBC): http://evbc.uni-jena.de/tools/coronavirus-tools/.

Tool	Advancing SARS-CoV-2 research by	License	Link(s)
**Detection and annotation**			
PriSeT	computing SARS-CoV-2 specific primers for RT-PCR tests	GPLv3	https://github.com/mariehoffmann/PriSeT
CoVPipe	reproducible, reliable and fast analysis of NGS data	GPLv3	https://gitlab.com/RKIBioinformatics Pipelines/ncov_minipipe
poreCov	reducing time-consuming bioinformatic bottlenecks in processing sequencing runs	GPLv3	https://github.com/replikation/poreCov
VADR	validation and annotation of SARS-CoV-2 sequences	public domain	https://github.com/nawrockie/vadr
V-Pipe	reproducible NGS-based end-to-end analysis of genomic diversity in intra-host virus populations	APLv2	https://cbg-ethz.github.io/V-pipe/ https://github.com/cbg-ethz/V-pipe
Haploflow	detection and full-length reconstruction of multi-strain infections	APLv2	https://github.com/hzi-bifo/Haploflow https://nextcloud.bifo.helmholtz-hzi.de/s/j4MyspJs5kfdZxy
VIRify	identifying viruses in clinical samples	APLv2	https://github.com/EBI-Metagenomics/emg-viral-pipeline
VBRC genome analysis tools	visualizing differences between coronavirus sequences at different levels of resolution	GPLv3	https://www.4virology.net
VIRULIGN	fast, codon-correct multiple sequence alignment and annotation of virus genomes	GPLv2	https://github.com/rega-cev/virulign
Rfam COVID-19	annotating structured RNAs in coronavirus sequences and predicting secondary structures	CC0	https://rfam.org/covid-19
UniProt COVID-19	providing latest knowledge on proteins relevant to the disease for virus and host	CC BY 4.0	https://covid-19.uniprot.org/
Pfam	protein detection and annotation for outbreak tracking and studying evolution	CC0	https://pfam.xfam.org
**Tracking, epidemiology and evolution**			
Covidex	fast and accurate subtypification of SARS-CoV-2 genomes	GPLv3	https://sourceforge.net/projects/covidex https://cacciabue.shinyapps.io/shiny2/
Pangolin	assigning a global lineage to query genomes	GPLv3	https://pangolin.cog-uk.io/ https://github.com/hCoV-2019/pangolin/
BEAST 2	understanding geographical origin, and evolutionary and transmission dynamics	LGPL	https://www.beast2.org/
Phylogeographic reconstruction	studying the global spread of the pandemic with particular focus on air transportation data	APLv2	https://github.com/hzi-bifo/Phylogeography_Paper
COPASI	modelling the dynamics of the epidemic and effect of interventions	Artistic License 2.0	http://copasi.org/ https://github.com/copasi
COVIDSIM	analysing effects of contact reduction measures and guide political decision making	—	http://www.kaderali.org:3838/covidsim
CoV-GLUE	tracking changes accumulating in the SARS-CoV-2 genome	(AGPLv3)[a]	http://cov-glue.cvr.gla.ac.uk/
PoSeiDon	detection of positive selection in protein-coding genes	MIT License	https://github.com/hoelzer/poseidon
**Drug design**			
VirHostNet	understanding molecular mechanisms underlying virus replication and pathogenesis	—[b]	http://virhostnet.prabi.fr/
CORDITE	carrying out meta-analyses on potential drugs and identifying potential drug candidates for clinical trials	CC BY-ND	https://cordite.mathematik.uni-marburg.de
CoVex	identifying already approved drugs that could be repurposed to treat COVID-19	—[c]	https://exbio.wzw.tum.de/covex/
P-HIPSTer	enabling the discovery of PPIs commonly employed within the coronavirus family and PPIs associated with their pathogenicity	—[d]	http://www.phipster.org/

[a]License of the underlying software system GLUE; [b]All data open access; [c]Source code available upon request; [d]Predictions available

pertise in deploying this technology in the sequencing and surveillance of outbreaks, including Zika and Ebola [11]. Nanopore sequencing is used to quickly generate high accuracy genomes of SARS-CoV-2 and track both transmission of COVID-19 and viral evolution over time (see `poreCov`).

In addition to amplicon-based sequencing approaches, metagenomic/-transcriptomic sequencing offers the ability to identify the primary pathogen and additional infections that may be present [12]. It can be used to identify coronaviruses in clinical and environmental samples, e.g., from human Bronchoalveolar lavage fluid (see `VIRify`). SARS-CoV-2 genomic traces in human faecal metagenomes from before the pandemic support the hypothesis of a possible presence of a most recent common ancestor of SARS-CoV-2 in the human population before the outbreak of the current pandemic, possibly in an inactive non-virulent form [13]. Further, metagenomics helps to check sequence divergence as the virus could undergo mutation and recombination with other human coronaviruses.

To help fight the COVID-19 pandemic, it is essential to make high-quality SARS-CoV-2 genome sequence data and metadata openly available through open databases either via a data-access agreement (e.g., GISAID[3]) or without restrictions (e.g., Genbank[4]). On GISAID (Global Initiative on Sharing All Influenza Data), laboratories around the world have shared viral genome sequence data with unprecedented speed[5]. **update numbers in footnote** We encourage researchersResearchers are encouraged to submit genome sequences to public databases that do not impose limitations on the sharing and use of the genomic sequences. NCBI offers a new streamlined submission process for SARS-CoV-2 data[6].

Several bioinformatics tools have been developed for the detection and annotation of SARS-CoV-2 genomes (see `VADR`, `V-Pipe`, `VIRify`, VBRC tools, `VIRULIGN`). Comparative genomics helps to detect differences to other coronaviruses, e.g., SARS-CoV-1, which might affect the functionality and pathogenesis of the virus.

Aside from coding sequences and proteins, the identification of conserved functional RNA secondary structures (see `Rfam`) is essential to understanding the molecular mechanisms of the virus life-cycle [14, 15]. Coronaviruses are known to have highly structured, conserved untranslated regions, which harbour cis-regulatory RNA secondary structure, controlling viral replication and translation, and even small changes in these structures reduce the viral load drastically [16–18].

[3] https://www.gisaid.org/

[4] https://submit.ncbi.nlm.nih.gov/sarscov2/

[5] >71,00030,000 SARS-CoV-2 genomic sequences on July 23May 22, 2020

[6] https://ncbiinsights.ncbi.nlm.nih.gov/2020/04/09/sars-cov2-data-streamlined-submission-rapid-turnaround/

Studying viral genomic diversity and the evolution of coding and non-coding sequences (see `UniProt`, `Pfam`, `Rfam`) is important for a better understanding of the evolution and epidemiology of SARS-CoV-2 (see Section **Tracking, epidemiology and evolution**), and the molecular mechanisms underlying COVID-19 pathogenesis (see Section **Drug design**).

2.1 PriSeT: Primer Search Tool

`PriSeT` [19] is a software tool that identifies chemically suitable PCR primers in a reference data set. The reference data set can be a FASTA file of complete genomes or a set of short regions. It is optimized for metabarcoding experiments where species are identified from an environmental sample based on a barcode – a relatively short region from the genome. The most frequently applied type of PCR for such experiments is the paired-end PCR – two different primer sequences are chosen to be complementary to the template and located within an offset range. The region in between is the amplicon or barcode and will be matched against the reference database to resolve operational taxonomic units to organisms. The precise constraint ranges can be adjusted by the user.

SARS-CoV-2 tests typically use mucus from the nose or throat that undergo a metabarcoding analysis. For DNA amplification RT-PCR is applied, which has more stringent requirements for the primer sequences and the DNA product length. Fig 1 shows the approximate locations of *in silico* transcripts of 114 primer pairs computed by PriSeT on 19 SARS-CoV-2 genomes with recommended RT-PCR settings [19]. The corresponding primer pairs have no co-occurrences in other coronaviruses. An additional online search on NCBI's GenBank confirmed that they also have no matches in any other sequences that are not assigned to SARS-CoV-2.`PriSeT` computes frequent k-mers that could serve as primer candidates, combines them to pairs, and ranks them by frequency and taxonomic coverage. When applied to SARS-CoV-2 genomes and adjusting the parameters to the ones of an RT-PCR, `PriSeT` computes primer pairs that occur in all genomes and are suitable for RT-PCR. These primer pairs can then be filtered further for those producing transcripts that have no matches outside the SARS-CoV-2 taxon. A list of SARS-CoV-2-specific primer pairs computed on 19 SARS-CoV-2 genomes is available on ResearchGate[7].

The computation of SARS-CoV-2-specific primers will help to design RT-PCR tests, since the resulting barcodes serve as unique identifiers for SARS-CoV-2 and avoid false-negative and false-positive identifications.

`PriSeT` is hosted on GitHub under the GNU General Public License v3.0 (GPLv3): https://github.com/mariehoffmann/PriSeT.

[7] https://www.researchgate.net/publication/340418344_Primer_pairs_for_detection_of_SARS-CoV-2_via_RT-PCR

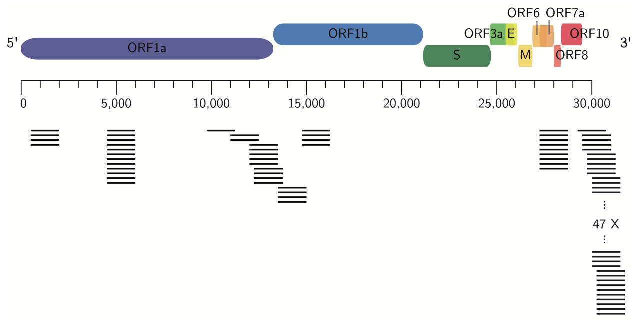

Figure 1: SARS-CoV-2-specific primers computed with `PriSeT`. Approximate amplicon locations of *de novo* computed primer pairs for SARS-CoV-2 with no co-occurrences in other genomes in GenBank (on April 03, 2020).

2.2 CoVPipe: Amplicon-based genome reconstruction

`CoVPipe` is a highly optimized and fully automated workflow for the reference-based reconstruction of SARS-CoV-2 genomes based on next-generation amplicon sequencing data using CleanPlex® SARS-CoV-2 panels (Paragon Genomics, Hayward, CA, USA) from swab samples. The pipeline applies read classification, clipping of raw reads to remove terminal PCR primer sequences or primer hybrids as well as Illumina adapters and low-quality bases. The processed reads are then aligned to a given reference sequence using `BWA-MEM` [20]. Resulting BAM files are evaluated to report mapping quality measurements like coverage, read depth, and insert size (`bedtools` v2.27 and `samtools` v1.3). Variants are called using `GATK` (v4.1) [21] and filtered following best practices of `GATK`. Finally, different consensus sequences can be created using different masking methods. Additionally, detailed information such as coverage, genomic localization and effect on respective gene products are reported for each variant site.
The pipeline is designed for reproducibility and scalability in order to ensure reliable and fast data analysis of SARS-CoV-2 data. The workflow itself is implemented using `Snakemake` [22], which provides advanced job balancing and input/output control mechanisms, and uses `conda` [23] to provide well defined and harmonized software environments.
`CoVPipe` is available via GitLab under GPLv3: https://gitlab.com/RKIBioinformaticsPipelines/ncov_minipipe.

2.3 poreCov: Rapid sample analysis for nanopore sequencing

Nanopore workflows were previously used in other outbreak situations, e.g., Zika, Ebola, Yellow Fever, Swine Flu, and can deliver a consensus viral genome after approximately seven hours[8]. The ARTIC network provides all the necessary information, tools, and protocols to assist groups in sequencing the coronavirus via nanopore sequencing[9]. These protocols utilize a multiplex PCR approach to amplify the virus directly from clinical samples, followed by sequencing and bioinformatic steps to assemble the data[10]. Due to the small viral genome, up to 24 samples can be sequenced at the same time. Rapid sample analysis is, therefore, of particular interest.
The workflow `poreCov` is implemented in `nextflow` [24] for full parallelization of the workload and stable sample processing (see Fig. 2). `poreCov` generates all necessary results and information before scientists continue to analyze their genomes or make them public on, e.g., GISAID or ENA / NCBI. The workflow carries out all necessary steps from basecalling to assembly depending on the user input, followed by lineage prediction of each genome using `Pangolin` (see Sec. 3.2). Furthermore, read coverage plots are provided for each genome to assess the amplification quality of the multiplex PCR. In addition, `poreCov` includes a quick time tree-based analysis of the inputs against reference sequences using `augur`[11] and `toytree`[12] for visualization. `poreCov` supports scientists in their SARS-CoV-2 research by reducing the time-consuming bioinformatic bottlenecks in processing dozens of SARS-CoV-2 sequencing runs.
All tools are provided via 'containers' (pre-build and stored on docker hub) to generate a reproducible workflow in various working environments. `poreCov` is ~~freely~~ available on GitHub under GPLv3: https://github.com/replikation/poreCov.

2.4 VADR: SARS-CoV-2 genome annotation and validation

`VADR` validates and annotates viral sequences based on models built from reference sequences [25]. Coronavirus models, based on NCBI RefSeq [26] entries, including one for SARS-CoV-2 (NC_045512.2), are available for analyzing coronavirus sequences. `VADR` computes an alignment of each incoming sequence against the RefSeq and uses it to map the RefSeq features, which include protein coding sequences (CDS), genes, mature peptides (`mat_peptide`), and structural RNA (`stem_loop`) features. The ORF1ab polyprotein CDS involves a programmed ribosomal frameshift, which `VADR` is capable of properly annotating. The tool identifies and outputs information about more than 40 types of problems with sequences, such as early stop codons in CDS, and has been in use by GenBank for screening and annotating incoming SARS-CoV-2 sequence submissions since March 2020. `VADR` (v1.1) includes heuristics for accelerating annotation and for dealing with stretches of ambiguous N nucleotides, that were specifically added for SARS-CoV-2 analysis.

[8] https://nanoporetech.com/about-us/news/novel-coronavirus-covid-19-information-and-updates

[9] https://artic.network/ncov-2019

[10] https://artic.network/ncov-2019/ncov2019-bioinformatics-sop.html

[11] https://github.com/nextstrain/augur

[12] https://github.com/eaton-lab/toytree

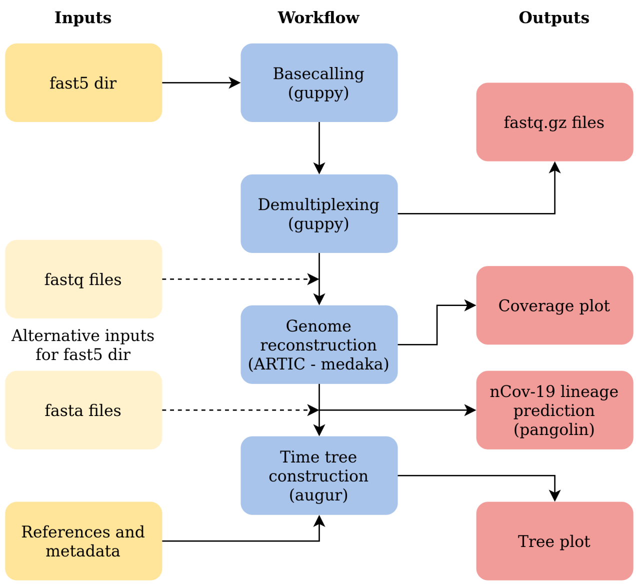

Figure 2: Simplified overview of the `poreCov` workflow. The individual workflow steps (blue) are executed automatically depending on the input (yellow). Instead of using raw nanopore fast5 files, fastq files or complete SARS-CoV-2 genomes can be used as an alternative input. If reference genomes and location/times are added, a time tree is additionally constructed.

`VADR` helps advance SARS-CoV-2 research by standardizing the annotation of SARS-CoV-2 sequences deposited in GenBank and other databases and by allowing researchers to fully annotate and screen their sequences for errors due to misassembly or other problems.
`VADR` is ~~freely~~ available via GitHub (public domain): https://github.com/nawrockie/vadr including specific instructions for use on SARS-CoV-2 sequences[13].

2.5 V-Pipe: Calling single-nucleotide variants and viral haplotypes

`V-pipe` [27] is a bioinformatics pipeline that integrates various computational tools for the analysis of viral high-throughput sequencing data. It supports the reproducible end-to-end analysis of intra-host NGS data, including quality control, read mapping and alignment, and inference of viral genomic diversity on the level of both single-nucleotide variants (SNVs) and long-range viral haplotypes. `V-pipe` uses the workflow management system `Snakemake` [22] to organize the order of required computational steps, and it supports cluster computing environments. It is easy to use from the command line, and `conda` [23] environments facilitate installation. `V-pipe`'s modular architecture allows users to design their pipelines and developers to test their tools in a defined environment, enabling best practices for viral bioinformatics.
A recent release of `V-pipe` addresses specifically the analysis of SARS-CoV-2 sequencing data. It uses the strain NC_045512 (GenBank: MN908947.3) as the default for read mapping and reporting of genetic variants, and it includes several improvements, for example, for calling single-nucleotide variants. Also, `V-pipe` can generate a comprehensive and intuitive visualization of the detected genomic variation in the context of various annotations of the SARS-CoV-2 genome. This summary of the output can help to generate diagnostic reports based on viral genomic data.
`V-pipe` is an SIB resource[14] and available via GitHub under the Apache License 2.0 (APLv2): https://github.com/cbg-ethz/V-pipe. Users are supported through the website[15], tutorials, videos, a mailing list, and the dedicated wiki pages of the GitHub repository.

2.6 Haploflow: Multi-strain aware *de novo* assembly

Viral infections often include multiple related viral strains [28], either due to co-infection or within-host evolution. These strains - haplotypes - may vary in phenotype due to certain, strain-specific genetic properties [29]. It is not entirely clear yet whether SARS-CoV-2 has a tendency for multiple infections, though there are indications that co-infections with other Coronaviruses do occur [30]. Most assemblers struggle with resolving complete viral haplotypes, even though these may be critical for the choice of therapy. `Haploflow` is a novel, de Bruijn graph-based assembler for the *de novo*, strain-resolved assembly of viruses that is able to rapidly resolve differences up to a base-pair level between two viral strains. `Haploflow` will help advance SARS-CoV-2 research by enabling the detection and full-length reconstruction of SARS-CoV-2 multi-strain infections.
`Haploflow` is available on GitHub under APLv2: https://github.com/hzi-bifo/Haploflow~~is freely available via https://nextcloud.bifo.helmholtz-hzi.de/s/j4MyspJs5kfdZxy~~

2.7 VIRify: Annotation of viruses in meta-omic data

`VIRify` is a recently developed, generic pipeline for the detection, annotation, and taxonomic classification of viral and phage contigs in metagenomic and metatranscriptomic assemblies. This pipeline is part of the repertoire of analysis services offered by `MGnify` [31]. `VIRify`'s taxonomic classification relies on the detection of taxon-specific profile hidden Markov models (HMMs), built upon a set of 22,014 orthologous protein domains and referred to as `ViPhOGs`. Included in this profile HMM database are 139 models that serve as specific markers for taxa within the *Coronaviridae* family.
Here, we show the applicability of `VIRify` on the assembly of a metatranscriptomic dataset from a human

[13] https://github.com/nawrockie/vadr/wiki/Coronavirus-annotation

[14] https://www.sib.swiss/research-infrastructure/database-software-tools/sib-resources

[15] https://cbg-ethz.github.io/V-pipe/

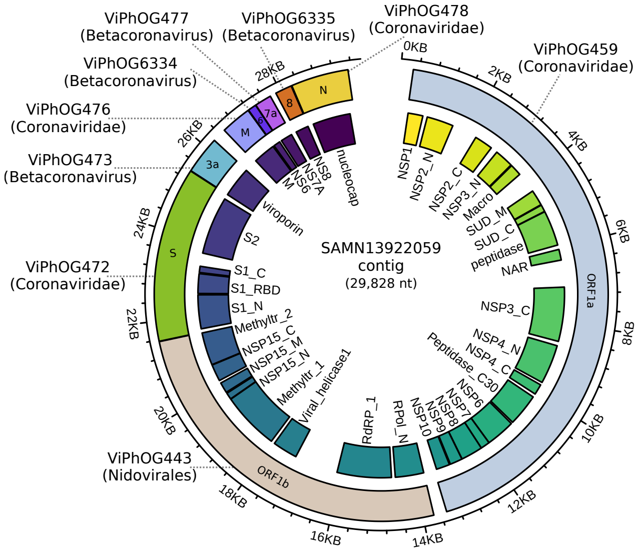

Figure 3: Sequence reads from a human lung metatranscriptome (sample accession: SAMN13922059) were first quality-filtered using `TrimGalore` v0.6.0 and subsequently assembled using `MEGAHIT` v1.1.3 [32] with default parameters. The resulting metatranscriptome assembly was processed through the `VIRify` pipeline. Based on the hits against the `ViPhOG` database, a 29 kb contig was classified as *Coronaviridae*. Functional protein domain annotations (inner track) were assigned by an `hmmsearch` v3.1b2 against Coronavirus models in `Pfam`. The image was created with `circlize` [33] and polished with Inkscape.

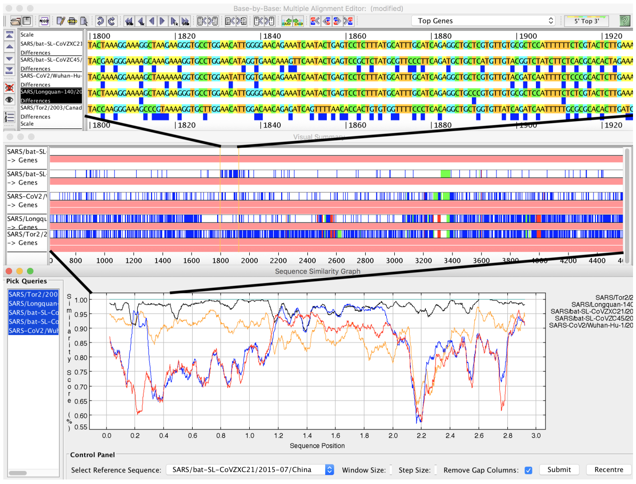

Figure 4: A region of recombination in coronavirus genomes at three levels of resolution in `Base-By-Base`. Top panel: aligned genomes; blue boxes show differences compared to top sequence in alignment. Middle panel: summary view showing differences and indels compared top sequence. Bottom panel: similarity plot comparing five genomes.

Bronchoalveolar lavage fluid. Within this assembly, a 29 kb contig was classified by `VIRify` as belonging to the *Coronaviridae* family (see Fig. 3). This shows the utility of the `VIRify` pipeline, used in isolation from `MGnify`, for studying coronaviruses in the human respiratory microbiome.

`VIRify` can be used for the identification of coronaviruses in clinical and environmental samples. Due to the intrinsic differences between metatranscriptomes and metagenomes, additional considerations regarding quality control, assembly, post-processing and classification have to be kept in mind[16].

`VIRify` is available via GitHub under APLv2: https://github.com/EBI-Metagenomics/emg-viral-pipeline.

2.8 Genome analysis tools by VBRC

The Viral Bioinformatics Research Centre (`VBRC`) is a mature resource built specifically for virologists to facilitate the comparative analysis of viral genomes. Within `VBRC`, a `MySQL` database created from GenBank files supports numerous analysis tools. The curated database is accessed through `Virus Orthologous Clusters` [34], a powerful, but easy-to-use database GUI. `Base-By-Base` [35–37] is a tool for generating, visualizing and editing multiple sequence alignments. It can compare genomes, genes or proteins via alignments and plots. Users can add comments to sequences and save alignments to a local computer. `Viral Genome Organizer` [38] visualizes and compares the organization of genes within multiple complete viral genomes. The tool allows the user to export protein or DNA sequences and can display START/STOP codons for 6-frames as well as open reading frames and other user-defined results. If genomes are loaded from the database, it can display shared orthologs. `Genome Annotation Transfer Utility` [39] is a tool for annotating genomes using information from a reference genome. It provides for interactive annotation, automatically annotating genes that are very similar to the reference virus but leaving others for a human decision.

The `VBRC` was developed for dsDNA viruses but has been adapted for coronaviruses. SARS-CoV-2 and closely related viruses have been added to the database. `VBRC` tools will help to visualize differences between coronavirus sequences at different levels of resolution (see Fig. 4).

`VBRC` is available via https://www.4virology.net; all tools are published under GPLv3.

2.9 VIRULIGN: Codon-correct multiple sequence alignments

`VIRULIGN` was developed for fast, codon-correct multiple sequence alignment and annotation of virus genomes, guided by a reference sequence [40]. A codon-aware alignment is essential for studying the evolution of coding nucleotide sequences to aid vaccine and antiviral development [41], to understand the emergence of drug resistance [42] and to quantify epi-

[16] for details, see https://github.com/EBI-Metagenomics/emg-viral-pipeline

demiological potential [43]. Theys et al. [44] have shown that a representative and curated annotation of open reading frames and proteins is essential to study emerging pathogens. To this end, a SARS-CoV-2 reference sequence and genome annotation have been added to VIRULIGN, based on the first available genome sequence [1], covering all reading frames and proteins.

VIRULIGN is easy to install, enabling scientists to perform large-scale analyses on their local computational infrastructure. VIRULIGN is particularly well suited to study the rapidly growing number of SARS-CoV-2 genomes made available [45], due to its efficient alignment algorithm that has linear computational complexity with respect to the number of sequences studied. Furthermore, VIRULIGN's flexible output formats (e.g., CSV file with headers corresponding to the genome annotation) facilitate its integration into analysis workflows, lowering the threshold for scientists to deliver advanced bioinformatics pipelines [46, 47] and databases [48], that are necessary to track the COVID-19 pandemic.

VIRULIGN is available via GitHub under the the GNU General Public License v2.0 (GPLv2): https://github.com/rega-cev/virulign.

2.10 Rfam COVID-19 Resources: Coronavirus-specific RNA families

Rfam [49] is a database of RNA families that hosts curated multiple sequence alignments and covariance models. To facilitate the analysis of Coronavirus sequences, Rfam produced a special release 14.2 with ten new families representing the entire 5' and 3' untranslated regions (UTRs) from *Alpha*-, *Beta*-, *Gamma*-, and *Deltacoronaviruses*. A specialised set of *Sarbecovirus* models is also provided, which includes SARS-CoV-1 and SARS-CoV-2 sequences. The families are based on a set of high-quality whole genome alignments that have been reviewed by expert virologists. In addition, Rfam now contains a revised set of non-UTR Coronavirus structured RNAs, such as the frameshift stimulating element, s2m RNA, and the 3' UTR pseudoknot.

The new Rfam families can be used in conjunction with the Infernal software [50] to annotate structured RNAs in Coronavirus sequences and predict their secondary structure (see Fig. 5). Table 2 shows the results for the SARS-CoV-2 RefSeq entry (NC_045512.2). In addition, the online Rfam sequence search enables users to scan genomic sequences and find the RNA elements.

The Coronavirus Rfam families are available freely available under the Creative Commons Zero (CC0) licence at https://rfam.org/covid-19.

2.11 UniProt COVID-19 protein portal: rapid access to protein information

UniProt [51] has recognised the urgency of annotating and providing access to the latest information on proteins relevant to the disease for both the virus and human host. In response, the COVID-19 UniProt portal provides early pre-release access to (i) SARS-CoV-2 annotated protein sequences, (ii) closest SARS proteins from SARS 2003, (iii) human proteins relevant to the biology of viral infection, like receptors and enzymes, (iv) ProtVista [52] visualisation of sequence features for each protein, (v) links to sequence analysis tools, (vi) access to collated community-contributed publications relevant to COVID-19, as well as (vii) links to relevant resources.

The COVID-19 portal enables community crowdsourcing of publications via the "Add a publication" feature within any entry. Thus, the community can assist in associating new or missing publications to relevant UniProt entries. ORCID is used as a mechanism to validate user credentials as well as recognition for contribution. Ten publication submissions have been received so far, contributing to our understanding of the virus biology. The COVID-19 UniProt portal advances SARS-CoV-2 research by providing latest knowledge on proteins relevant to the disease for both the virus and human host.

The COVID-19 UniProt portal is available under the Creative Commons Attribution License (CC BY 4.0) via https://covid-19.uniprot.org/. UniProt also hosted webinars to describe the portal[17] and publication submission system[18].

2.12 Pfam protein families database

The Pfam protein families database is widely used in the field of molecular biology for large-scale functional annotation of proteins [53]. The latest release of Pfam, version 33.1, contains an updated set of models that comprehensively cover the proteins encoded by SARS-CoV-2 (see Table 3). The only SARS-CoV-2 protein that lacks a match is Orf10, a small putative protein found at the 3'-end of the SARS-CoV-2 genome, which appears to lack similarity to any other sequence in UniProtKB[19]. The Pfam profile hidden Markov model (HMM) library in combination with the HMMER software [54] facilitates rapid search and annotation of coronaviruses and can be used to generate multiple sequence alignments that allow the identification of mutations and clusters of related sequences, particularly useful for outbreak tracking and studying the evolution of coronaviruses.

The Pfam HMM library can be downloaded from https://pfam.xfam.org and can be used in combination with pfam_scan to perform Pfam analysis locally. Multiple sequence alignments of matches can be generated using hmmalign[20]. Precalculated matches and alignments are available from the Pfam FTP site[21].

[17] https://www.youtube.com/watch?v=EY69TjnVhRs
[18] https://www.youtube.com/watch?v=sOPZHLtQK9k
[19] https://covid-19.uniprot.org/
[20] http://hmmer.org/
[21] ftp://ftp.ebi.ac.uk/pub/databases/Pfam/releases/Pfam_SARS-CoV-2_2.0/

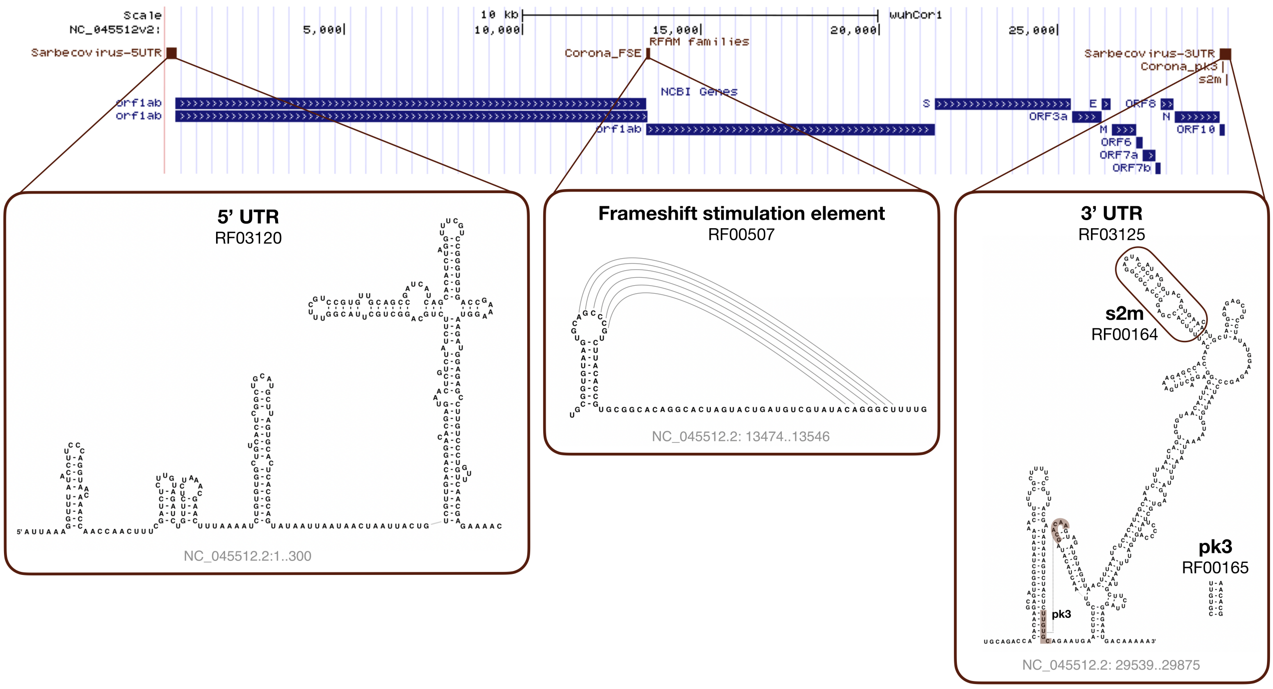

Figure 5: SARS-CoV-2 `Rfam` secondary structure predictions. The sequence is based on the NC_045512.2 RefSeq entry displayed with the wuhCor1 UCSC Genome Browser alongside the NCBI Genes track.

Table 2: `Rfam` version 14.2 matches to the SARS-CoV-2 RefSeq entry NC_045512.2

RefSeq coordinates	Rfam accession	Rfam ID	Rfam description	Comment
NC_045512.2/1-299	RF03120	Sarbecovirus-5UTR	Sarbecovirus 5' UTR	See Rfam family RF03117 for Betacoronavirus 5' UTR.
NC_045512.2/13,469-13,550	RF00507	Corona_FSE	Coronavirus frameshifting stimulation element	
NC_045512.2/29,536-29,870	RF03125	Sarbecovirus-3UTR	Sarbecovirus 3' UTR	See Rfam family RF03122 for Betacoronavirus 3' UTR.
NC_045512.2/29,603-29,662	RF00164	Corona_pk3	Coronavirus 3' UTR pseudoknot	The family annotates the pseudoknot found in the 3' UTR (RF03120).
NC_045512.2/29,727-29,769	RF00165	s2m	Coronavirus 3' stem-loop II-like motif (s2m)	The family is a subset of the 3' UTR model (RF03120) that corresponds to the PDB:1XJR 3D structure from SARS-CoV-1.

Pfam is freely available under the Creative Commons Zero (CC0) licence.

3 Tracking, epidemiology and evolution

As there is no universal approach for classifying a virus species' genetic diversity, the phylogenetic clades are referred to by different terms, such as 'subtypes', 'genotypes', or 'groups'. However, phylogenetic assignment is important for studies on virus epidemiology, evolution, and pathogenesis (see Covidex, Pangolin). Thus, a nomenclature system for naming the growing number of phylogenetic lineages that make up the population diversity of SARS-CoV-2 is needed. Rambaut et al. [45] have described a lineage nomenclature for SARS-CoV-2 that arises from a set of fundamental evolutionary, phylogenetic and epidemiological principles.

Phylodynamic models may aid in dating the origins of pandemics, provide insights into epidemiological parameters, e.g., R_0 [55], or help determine the effectiveness of virus control efforts (see BEAST 2, phylogeographic reconstruction). Phylodynamic analyses aim to conclude epidemiological processes from viral phylogenies, at the most basic level by comparing genetic relatedness to geographic relatedness.

Mathematical epidemiological models project the progress of the pandemic to show the likely outcome and help inform public health interventions (see COPASI, COVIDSIM). Such models help with analysing the effects of contact reduction measures or other interventions, forecasting hospital resource usage, and guiding political decision-making.

As the pandemic progresses, SARS-CoV-2 is naturally accumulating mutations. On average, the observed changes would be expected to have no or minimal consequence for virus biology. However, tracking these changes (see CoV-GLUE, PoSeiDon) will help us better understand the pandemic and could help improve the effectiveness of antiviral drugs and vaccines, both pharmaceutical prevention measures that will be crucial to control the COVID-19 pandemic [56, 57].

3.1 Covidex: Alignment-free subtyping using machine learning

Viral subtypes or clades represent clusters among isolates from the global population of a defined species. Subtypification is relevant for studies on virus epidemiology, evolution and pathogenesis. Most subtype classification methods require the alignment of the input data against a set of pre-defined subtype reference sequences. These methods can be computationally expensive, particularly for long sequences such as SARS-CoV-2 ($\approx$30 kb per genome). To tackle this problem, machine learning tools may be used for virus subtyping [58]. Covidex was developed as an open-source alignment-free machine learning subtyping tool. It is a shiny app [59] that allows fast and accurate (out-of-bag error rate $< 1.5\,\%$) classification of viral genomes in pre-defined clusters (see Fig. 6). For SARS-CoV-2, the default uploaded model is based on Nextstrain [60] and GISAID data [61]. Alternatively, user-uploaded models can be used. Covidex is based on a fast implementation of random forest trained over a k-mer database [62, 63]. By training the classification algorithms over k-mer frequency vectors, Covidex substantially reduces computational and time requirements and can classify hundreds of SARS-CoV-2 genomes in seconds. Thus, in the context of the current global pandemic where the number of available SARS-CoV-2 genomes is growing exponentially, SARS-CoV-2 research can benefit from this specific tool designed to reduce the time needed in data analysis significantly.

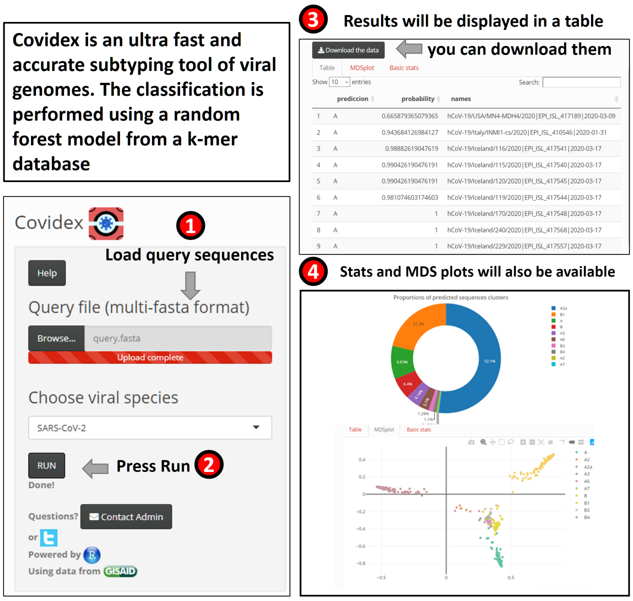

Figure 6: Overview of Covidex for viral subtyping analysis. Left: The user is expected to load a sequence file and to select the model that will be applied for classification. Models may be selected from the default list or uploaded by the user. Right: The program output (table and plots).

Covidex is available via SourceForge under GPLv3: https://sourceforge.net/projects/covidex or the web application https://cacciabue.shinyapps.io/shiny2/.

3.2 Pangolin: Phylogenetic Assignment of Named Global Outbreak LINeages

Pangolin assigns a global lineage to query SARS-CoV-2 genomes by estimating the most likely placement within a phylogenetic tree of representative sequences from all currently defined global SARS-CoV-2 lineages based on the lineage nomenclature proposed by Rambaut et al. [45]. It is easily scalable so that it can be run on either thousands or a handful of sequences. Internally, pangolin runs mafft [64] and iqtree [65, 66], providing a guide tree and alignment to keep analysis overhead relatively lightweight.

Table 3: Pfam **version 33.1 matches to the proteome of SARS-CoV-2 found in** UniProtKB**.**

Uniprot accession ID	Gene Name	Pfam accession	Pfam ID	Pfam description
sp|P0DTC1|R1A_SARS2	ORF1ab	PF11501	bCoV_NSP1	Betacoronavirus replicase NSP1
		PF19211	CoV_NSP2_N	Coronavirus replicase NSP2, N-terminal
		PF19212	CoV_NSP2_C	Coronavirus replicase NSP2, C-terminal
		PF12379	bCoV_NSP3_N	Betacoronavirus replicase NSP3, N-terminal
		PF01661	Macro	Macro domain
		PF11633	bCoV_SUD_M	Betacoronavirus single-stranded poly(A) binding domain
		PF12124	bCoV_SUD_C	Betacoronavirus SUD-C domain
		PF08715	CoV_peptidase	Coronavirus papain-like peptidase
		PF16251	bCoV_NAR	Betacoronavirus nucleic acid-binding (NAR)
		PF19218	CoV_NSP3_C	Coronavirus replicase NSP3, C-terminal
		PF19217	CoV_NSP4_N	Coronavirus replicase NSP4, N-terminal
		PF16348	CoV_NSP4_C	Coronavirus replicase NSP4, C-terminal
		PF05409	Peptidase_C30	Coronavirus endopeptidase C30
		PF19213	CoV_NSP6	Coronavirus replicase NSP6
		PF08716	CoV_NSP7	Coronavirus replicase NSP7
		PF08717	CoV_NSP8	Coronavirus replicase NSP8
		PF08710	CoV_NSP9	Coronavirus replicase NSP9
		PF09401	CoV_NSP10	Coronavirus RNA synthesis protein NSP10
sp|P0DTC2|SPIKE_SARS2	S	PF16451	bCoV_S1_N	Betacoronavirus-like spike glycoprotein S1, N-terminal
		PF09408	bCoV_S1_RBD	Betacoronavirus spike glycoprotein S1, receptor binding
		PF19209	CoV_S1_C	Coronavirus spike glycoprotein S1, C-terminal
		PF01601	CoV_S2	Coronavirus spike glycoprotein S2
sp|P0DTC3|AP3A_SARS2	ORF3a	PF11289	bCoV_viroporin	Betacoronavirus viroporin
sp|P0DTC4|VEMP_SARS2	E	PF02723	CoV_E	Coronavirus small envelope protein E
sp|P0DTC5|VME1_SARS2	M	PF01635	CoV_M	Coronavirus M matrix/glycoprotein
sp|P0DTC6|NS6_SARS2	ORF6	PF12133	bCoV_NS6	Betacoronavirus NS6 protein
sp|P0DTC7|NS7A_SARS2	ORF7a	PF08779	bCoV_NS7A	Betacoronavirus NS7A protein
sp|P0DTD8|NS7B_SARS	ORF7b	PF11395	bCoV_NS7B	Betacoronavirus NS7B protein
sp|P0DTC8|NS8_SARS2	ORF8	PF12093	bCoV_NS8	Betacoronavirus NS8 protein
sp|P0DTC9|NCAP_SARS2	N	PF00937	CoV_nucleocap	Coronavirus nucleocapsid
sp|P0DTD2|ORF9B_SARS2	ORF9b	PF09399	bCoV_lipid_BD	Betacoronavirus lipid binding protein
sp|P0DTD3|Y14_SARS2	ORF14	PF17635	bCoV_Orf14	Betacoronavirus uncharacterised protein 14 (SARS-CoV-2 like)

Pangolin has many applications, including frontline hospital use and local and global surveillance. For example, in hospitals sequencing SARS-CoV-2 samples, it could be used to rule out within-hospital transmission, informing infection control measures. It can also be used for surveillance purposes, summarising which lineages are present in an area of interest. The web-application also connects with Microreact[22] displaying query sequences in the context of the global lineages worldwide. pangolin is used as part of COG-UK's[23] data processing pipeline to assign lineages to UK sequences. Further, users can define their own finer-scale lineages, for instance within-country lineages, and provide their own guide tree and alignment.

Pangolin makes it easy to get useful information out of viral genome sequencing in real-time and can assist in identifying new introductions and in tracking the spread of SARS-CoV-2.

Pangolin is available ~~as command line tool~~ via GitHub under GPLv3: https://github.com/hCoV-2019/pangolin/ and as web application via https://pangolin.cog-uk.io/.

3.3 BEAST 2: Phylodynamics based on Bayesian inference

Important evolutionary and epidemiological questions regarding SARS-CoV-2 can be addressed using Bayesian phylodynamic inference [67], which allows the adequate combination of evidence from multiple independent sources of data, such as genome sequences, sampling dates and geographic locations. BEAST 2 [68] is an advanced computational software framework that enables sophisticated Bayesian analyses utilising a range of phylodynamic packages, e.g. [69–75]. The phylogenetic history (the tree) can be inferred simultaneously with evolutionary and epidemiological parameters, such that the uncertainty from all aspects of the joined model is accounted for and reflected in the results. Phylodynamic analysis of SARS-CoV-2 is crucial in understanding (i) SARS-CoV-2 evolutionary dynamics, particularly through estimation of the evolutionary rate at which mutations get fixated in the viral genome, (ii) the temporal origin of a selection of COVID-19 cases as an approximation of the time at which a sub-epidemic emerged, (iii) the geographical origin of sub-epidemics, (iv) SARS-CoV-2 transmission dynamics, e.g. through direct estimation of the effective reproduction number R_e and its changes through time, and (v) the proportion of undetected COVID-19 cases. Indeed, due to the evolutionary and epidemiological processes occurring on the same time scale, the diversity in the viral genome sheds light on between-host transmission dynamics - making Bayesian phylodynamic analysis of SARS-CoV-2 a crucial complement to classical epidemiological methods.

BEAST 2 is available via https://www.beast2.org/ under the GNU Lesser General Public License (LGPL).

3.4 Phylogeographic reconstruction using air transportation data

Phylogeographic methods combine genomic data with the sampling locations of viral isolates and models of spread, e.g. using air travel or local diffusion, to reconstruct the putative spread paths and outbreak origins of rapidly evolving pathogens. Reimering et al. [76] published a method that infers locations for internal nodes of a phylogenetic tree using a parsimonious reconstruction together with effective distances, as defined by Brockmann et al. [77]. Effective distances are calculated based on passenger flows between airports. A strong connection between two airports is represented by a small distance. Using these distances as a cost matrix, the parsimonious reconstruction identifies ancestral locations for internal nodes of the tree that minimize the distances along the phylogeny. This method allows rapid inferences of spread paths on a fine-grained geographical scale [76]. Reconstruction using effective distances infers phylogeographic spread more accurately than reconstruction using geographic distances or Bayesian reconstructions that do not use any distance information.

Phylogeographic reconstruction using air transportation data can be used to study the global spread of the SARS-CoV-2 pandemic, especially in the early phases when air travel still substantially contributed to the spread of the virus. The method is currently adapted to consider both air travel and local movement data within countries during inference to reflect the changing worldwide movements in different phases of the pandemic.

The code is included in the GitHub repository for Reimering et al. [76] under APLv2: https://github.com/hzi-bifo/Phylogeography_Paper

3.5 COPASI: Modeling SARS-CoV-2 dynamics with differential equations

COPASI is a dynamics simulator, originally focused on chemical and biochemical reaction networks [78]. However, it is by now also widely applied to other fields, including epidemiology. It allows simulating models with the traditional differential equation approach that represents populations as continua, as well as with a stochastic kinetics approach which considers populations are composed of individuals. COPASI has a common model representation for both these approaches, which allows switching between them with ease. Additionally, one can add arbitrary discrete events to models. This software is equipped with several algorithms that provide comprehensive analyses of models, and it has support for parameter estimation using a series of optimisation algorithms. COPASI has been used to model various aspects of virology, including mechanisms of action [79–82], phar-

[22] microreact.org

[23] https://www.cogconsortium.uk/

maceutical interventions [83], virus life-cycle [84], vaccine design [85] and dynamics of epidemics [86–88]. COPASI has also been applied to COVID-19, particularly to model the dynamics of the epidemic and effect of interventions [89]. Some of the authors have also used COPASI to model the local epidemics and forecast usage of hospital resources (P. Mendes) and to compare the possible advantages of contact network agent-based models over differential equation models (S. Hoops).

COPASI is available from http://copasi.org/ and https://github.com/copasi under the Artistic License 2.0.

3.6 COVIDSIM: Epidemiological models of viral spread

Classical epidemiological models have seen broad reuse in describing the COVID-19 outbreak. Deterministic or compartmental mathematical models assign individuals in a population to different subgroups and describe their dynamic changes using systems of differential equations. For SARS-CoV-2, the SEIR model and extended versions thereof are frequently used. The underlying model framework is not new at all, and related models have been described already at the beginning of the 20th century to model infectious diseases [90]. In brief, in the SEIR or SEIRD-Model, individuals in a population are grouped into *Susceptible* (S), *Exposed* (E), *Infected* (I), *Recovered* (R) and *Deceased* (D) individuals. Initially, all individuals except for a small number who are already infected are considered susceptible to infection. The model can then simulate the population infection dynamics, using parameters such as the incubation time or the average disease duration for parameterization of the differential equations. Such SEIR models have been used to predict the COVID-19 dynamics, e.g. in Spain and Italy, and to analyse the effect of control strategies [91]. Extended versions of the SEIR model were developed to guide political decision making [92]. For example, in Germany, this model is implemented in COVIDSIM, including hospitalised patients and patients in intensive care and implementing effects of contact reduction measures. It can be overlaid with data from different German federal states and data from other countries.~~We recently implemented a version of the model in COVIDSIM, a simulator which includes hospitalised patients and patients in intensive care and implements effects of contact reduction measures, and that can be overlaid with data from different German federal states and data from other countries.~~ This model has a convenient web interface (see Fig. 7), permitting the user to change model parameters and get an intuitive feeling for the model dynamics – allowing it to estimate infection parameters and to analyse effects of contact reduction measures and guide political decision making.

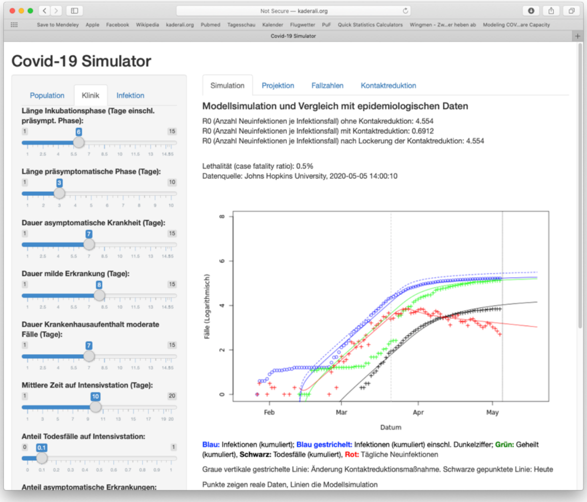

Figure 7: Web interface of the COVIDSIM simulator. The interface is allowing the user to modify model parameters and compare simulated dynamics with real infection data.

The web interface is ~~in German and~~ available via http://www.kaderali.org:3838/covidsim.

3.7 CoV-GLUE: tracking nucleotide changes in the SARS-CoV-2 genome

SARS-CoV-2 is naturally accumulating nucleotide mutations in its RNA genome as the pandemic progresses. Point mutations, specifically non-synonymous substitutions, will result in amino acid replacements in viral genome sequences, while other mutations will result in insertions or deletions (indels). On average the observed changes would be expected to have no or minimal consequence for virus biology. However tracking these changes will help us better understand and control the pandemic as mutations could arise with impact on virus biology and could lead to escape from antiviral drugs and future vaccines. The purpose of CoV-GLUE is to track the changes accumulating in the SARS-CoV-2 genome (see Fig. 8). The resource was developed exploiting GLUE, a data-centric bioinformatics environment for virus sequence data, with a focus on variation, evolution and sequence interpretation [93]. Sequences are downloaded from GISAID EpiCoV [94] approximately every week and added to a constrained alignment within the GLUE framework. Users can browse the accumulating variation or submit a FASTA file of a novel genome to CoV-GLUE for comparison to the available data. An amino acid replacements, indels and diagnostic primer design report is generated from the submitted data. The user can access the detected variants and using a phylogenetic placement maximum-likelihood method [95] visualise their sequence relative to a reference data set. The user's sequence is also assigned to a lineage consistent with Rambaut et al. [45].

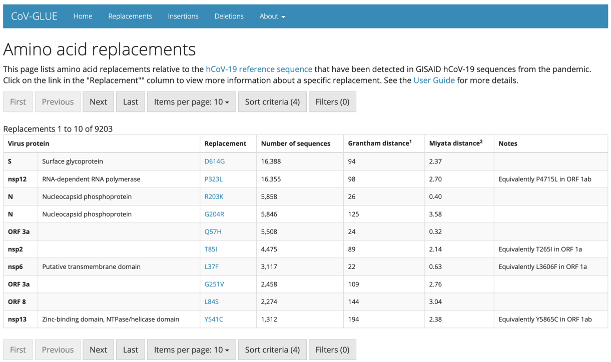

CoV-GLUE Home Replacements Insertions Deletions About

Amino acid replacements

This page lists amino acid replacements relative to the hCoV-19 reference sequence that have been detected in GISAID hCoV-19 sequences from the pandemic. Click on the link in the "Replacement"" column to view more information about a specific replacement. See the User Guide for more details.

First Previous Next Last Items per page: 10 Sort criteria (4) Filters (0)

Replacements 1 to 10 of 9203

Virus protein		Replacement	Number of sequences	Grantham distance[1]	Miyata distance[2]	Notes
**S**	Surface glycoprotein	D614G	16,388	94	2.37	
**nsp12**	RNA-dependent RNA polymerase	P323L	16,355	98	2.70	Equivalently P4715L in ORF 1ab
**N**	Nucleocapsid phosphoprotein	R203K	5,858	26	0.40	
**N**	Nucleocapsid phosphoprotein	G204R	5,846	125	3.58	
**ORF 3a**		Q57H	5,508	24	0.32	
**nsp2**		T85I	4,475	89	2.14	Equivalently T265I in ORF 1a
**nsp6**	Putative transmembrane domain	L37F	3,117	22	0.63	Equivalently L3606F in ORF 1a
**ORF 3a**		G251V	2,458	109	2.76	
**ORF 8**		L84S	2,274	144	3.04	
**nsp13**	Zinc-binding domain, NTPase/helicase domain	Y541C	1,312	194	2.38	Equivalently Y5865C in ORF 1ab

First Previous Next Last Items per page: 10 Sort criteria (4) Filters (0)

Figure 8: List of amino acid replacements to the SARS-CoV-2 reference sequence. Replacements have been detected in `GISAID` SARS-CoV-2 sequences from the pandemic using `CoV-GLUE`.

`CoV-GLUE` will help advance SARS-CoV-2 research by tracking changes accumulating in the SARS-CoV-2 genome. `CoV-GLUE` web application is available online via http://cov-glue.cvr.gla.ac.uk/. CoV-GLUE is not released as an open source installable GLUE package due to the legal restrictions on GISAID data (see Sec. 2). The underlying software system GLUE is open source and licensed under the GNU Affero General Public License v3.0 (AGPLv3).

3.8 PoSeiDon: Positive Selection Detection and Recombination Analysis

Viruses and their hosts are in constant competition, and selection pressure continuously affects the evolution of their genes. Selection pressure, in the form of positive selection, can be studied by comparing the rates of non-synonymous (dN) and synonymous substitutions (dS) in an alignment of orthologous genes. Over several sites (codons), the dN/dS ratio can reach values well above 1 [96]. Such positively selected sites are described in recent SARS-CoV-2 studies. For example, Velazquez-Salinas et al. [97] showed that the selection pressure on ORF3a and ORF8 genes can drive the evolution of the virus during the COVID-19 pandemic, while Korber et al. [98] describe worrying changes in the spike protein through the detection of positive selection.

`PoSeiDon` simplifies the detection of positive selection in protein-coding sequences [99]. Firstly, the pipeline builds a multiple sequence alignment, estimates a best-fitting substitution model, and performs a recombination analysis followed by the construction of all corresponding phylogenies. Secondly, positively selected sites under varying models are detected. The results are summarised in a user-friendly web page, providing all intermediate results and graphically displaying recombination events and positively selected sites.

The rapid detection of positive selection helps to monitor protein changes of SARS-CoV-2 during the pandemic. It provides potential target sites for drug development, helping to counteract the virus during its ”arms race” with the human species.

`Poseidon` is available via GitHub under MIT License: https://github.com/hoelzer/poseidon.

4 Drug design

To limit the pandemic threat, it is of utmost importance to develop therapy and vaccination strategies against COVID-19. Understanding the molecular mechanisms underlying the disease's pathogenesis is key to identifying potential drug candidates for clinical trials. Viral-host protein-protein interactions (PPIs) play a crucial role during viral infection and hold promising therapeutic prospects.

To facilitate the identification of potential drugs, a screening of known drugs and PPIs, referred to as drug repurposing, is usually cheaper and more time-efficient than designing drugs from scratch [100, 101]. This is especially true for SARS-CoV-2, as it is a member of a viral genus that has been thoroughly studied. Therefore, we can infer information and potential drug targets from other *betacoronaviruses*, and especially SARS-CoV-1. The described databases contain information about virus-host PPIs (see `VirHostNet`, `CoVex`) and virus-drug interactions (see `CORDITE`, `CoVex`) and gather information from other viruses and drugs to infer potential PPIs for SARS-CoV-2 (see `CoVex`, `P-HIPSTer`).

4.1 VirHostNet SARS-CoV-2 release

The complete understanding of molecular interactions between SARS-CoV-2 and host cellular proteins is key to highlight functions that are essential for viral replication and pathogenesis of COVID-19 outbreak. Toward this end, `VirHostNet` [102] was upgraded in March 2020 to include a comprehensive collection of protein-protein interactions manually annotated from the literature involving ORFeomes from multiple coronaviruses, including MERS-CoV, SARS-CoV-1 and SARS-CoV-2. This biocuration effort also incorporated, in close to real-time, the data obtained through affinity-purification mass spectrometry by the Korgan laboratory [103]. Hence, in a few days, more than 650 binary protein-protein interactions were made available to scientists working on COVID-19.

The `VirHostNet` resource was rapidly catalogued as a fair and open data resource to help fight against COVID-19 [104]. To leverage the cost of highly expensive experiments, open access is provided to the interology web application allowing fast and reproducible *in silico* prediction of SARS-CoV-2/human interactome ~~As a proof of concept, `VirHostNet` was used as a gateway to explore systems-level links between the SARS-CoV-2 proteins and host pathways involving apoptosis, autophagy and immune response [105].~~ The interactome predicted for SARS-CoV-2 was wired to an anti-apoptotic switch regulated by Bcl-2 family members that could potentially be a

therapeutic target. The network reconstruction identified the prosurvival protein Bcl-xL and the autophagy effector Beclin 1 as vulnerable nodes in the host cellular defense system against SARS-CoV-2. Interestingly, both proteins harbour a so-called Bcl-2 homology 3 (BH3)-like motif, which is involved in homotypic (inside the Bcl-2 family) and heterotypic interactions with other domains.
The `VirHostNet` SARS-CoV-2 release will accelerate research on the molecular mechanisms underlying virus replication as well as COVID-19 pathogenesis and will provide a systems virology framework for prioritizing drug candidates repurposing.
`VirHostNet` web application is available via http://virhostnet.prabi.fr/. All data is open access.

4.2 CORDITE: COROna Drug InTERactions database

`CORDITE` collects data on potential drugs, targets, and their interactions for SARS-CoV-2 from published articles and preprints [106]. `CORDITE` integrates many functionalities to enable users to access, sort, and download relevant data to conduct meta-analyses, to design new clinical trials, or even to conduct a curated literature search. `CORDITE` automatically incorporates publications from PubMed[24], bioRxiv[25], chemRxiv[26], and medRxiv[27] that report information on computational, *in vitro*, or case studies on potential drugs for COVID-19. Besides original research, reviews and comments are also included in the database. The information from the articles and preprints are manually curated by moderators and can be accessed via the web server or the open API. Moreover, registered clinical trials from the NIH[28] for COVID-19 are also included. Users can directly access the publications, interactions, drugs, targets, and clinical trials, and thus, the data can be easily integrated into other software or apps.
The `CORDITE` database is updated weekly and, at the date of submission, provides data for more than 700 interactions of 23 targets for more than 530 drugs from almost 300 publications and more than 240 clinical trials (as of May 19, 2020). It is thus the largest, curated database available for drug interactions for SARS-CoV-2. It allows researchers to carry out meta-analyses on potential drugs systematically and to identify potential drug candidates for clinical trials.
`CORDITE` can be accessed via
https://cordite.mathematik.uni-marburg.de (CC BY-ND).

4.3 CoVex: CoronaVirus Explorer

`CoVex` [107] is a network and systems medicine web platform that integrates experimental virus-human protein interactions for SARS-CoV-2 [103] and SARS-CoV-1 [102, 108], human protein-protein interactions [109] and drug-protein interactions [110–115] into a large-scale interactome (see Fig. 9). It allows biomedical and clinical researchers to predict novel drug targets as well as drug repurposing candidates using several state-of-the-art graph analysis methods specifically tailored to the network medicine context. Here, expert knowledge about virus replication, immune-related biological processes or drug mechanisms can be applied to compile a set of host or viral proteins (referred to as seeds). Alternatively, users can upload a list of proteins (e.g. differentially expressed genes, a list of proteins related to a molecular mechanism of interest) or proteins targeted by drugs of interest (e.g. a set of drugs known to be effective) as seeds to guide the analysis. Based on the selected seeds, `CoVex` offers three main actions: (1) searching the human interactome for viable drug targets, (2) identifying repurposable drug candidates, and (3) a combination of actions, i.e. starting from a selection of virus or virus-interacting proteins, users can mine the interactome for suitable drug targets for which, in turn, suitable drugs are identified. In summary, `CoVex` allows researchers to systematically identify already approved drugs that could be repurposed to treat SARS-CoV-2, which is faster than developing new drugs from scratch.
`CoVex` web application is available via
https://exbio.wzw.tum.de/covex/.

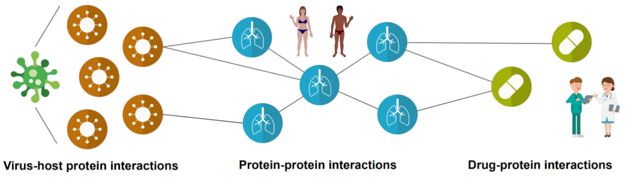

Figure 9: CoVex: CoronaVirus Explorer. `CoVex` is a network medicine web platform that allows its users to interactively mine a large interactome that integrates information about virus-host protein-interactions, known human protein-protein interactions as well as drug-protein interactions. `CoVex` can be used for identifying potential drug targets and drug repurposing candidates.

4.4 P-HIPSTer: a virus-host protein-protein interaction resource

Viral-host protein-protein interactions (PPIs) play a crucial role during viral infection by co-opting host cellular processes and hold promising therapeutic prospects. Along these lines, the `P-HIPSTer` database can significantly contribute to SARS-CoV2 research by providing: (1) testable hypotheses on molecular interactions underlying viral infection and pathogenesis and; (2) highlighting host factors and pathways that serve as potential drug targets to treat infection caused by different coronaviruses.
`P-HIPSTer` comprises ∼282,000 predicted viral-

[24] https://www.ncbi.nlm.nih.gov/pubmed/
[25] https://www.biorxiv.org/
[26] https://www.chemrxiv.org/
[27] https://www.medrxiv.org/
[28] https://clinicaltrials.gov/

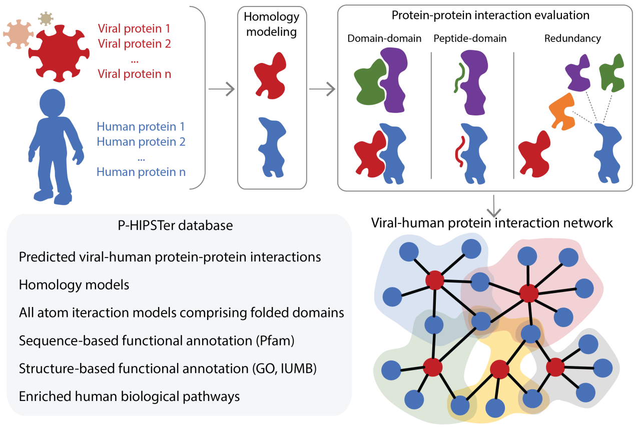

Figure 10: `P-HIPSTer` **combines sequence and structural information to predict viral-host PPIs**. `P-HIPSTer` evaluates the likelihood ratio (LR) for the potential interaction between a viral protein (in red) and a human protein (in blue) combining three evidences: i) Domain-domain LR that two structure domains interact based on known complex (green and purple domain-domain complex) comprised of their structural neighbours; ii) Peptide-domain LR that an unstructured peptide in one query binds to a structured domain in the second query based on known binding motifs/peptide-domain complex (green and purple peptide-domain complex) using both sequence and structural similarity iii) Redundancy LR based on evidence that multiple structural neighbours (in orange, purple and green) of one query protein is known to interact with the remaining query protein. Each viral protein is functionally annotated based on sequence and structural similarity (either using homology models or known protein structures) and their corresponding set of predicted interacting human proteins.

human PPIs on ~1,000 viruses with an experimental validation rate of ~76% [116]. Its predictive algorithm is an adaptation of `PrePPI` [117, 118] and combines sequence and structural information to infer viral-human PPIs mediated by domain-domain or peptide-domain contacts (see Fig. 10). In addition, `P-HIPSTer` builds all-atom interaction models for high-confidence PPI predictions involving folded domains and integrates sequence- and structure-based functional annotations for viral proteins at multiple levels, including host biological pathways based on the predicted PPIs [119–122]. Hence, `P-HIPSTer` constitutes a complimentary resource to high-throughput experimental approaches [103]. As of April 2020, `P-HIPSTer` contains predictions for 15 coronaviruses with varying pathogenic potential (alpha- and betacoronaviruses) and reports 4,587 viral-host PPIs involving 397 human proteins. This unique collection of predicted viral-human PPIs enables the discovery of PPIs commonly employed within the *Coronaviridae* family and PPIs associated with their pathogenicity.

The database is available via http://www.phipster.org/

5 Concluding remarks

Bioinformaticians around the world have reacted quickly to the COVID-19 pandemic by providing coronavirus-specific tools to advance SARS-CoV-2 research and boost the detection, understanding, and treatment of COVID-19. This review does not claim to be complete, and in light of the rapid ongoing research, further tools will be developed.

Efficient response to the pandemic requires high-quality SARS-CoV-2 data and meta-data [123] and newly released software to be available freely and as open source. Open source code invites other developers to improve the software. Preferably, code should be shared via a suitable repository such as GitHub, allowing for transparency and managing versioning and feature development. In particular, when software and resources are evolving as fast as the virus, versioning and reproducibility of all steps are of increasing importance. In the context of pipelines, versions of third-party tools should be fixed using package managers like Conda or by encapsulation using container software (Docker [124], Singularity). Workflow management systems such as SnakeMake or Nextflow [24, 125] allow easy installation and reproducible execution on various platforms. In the best case, all tools and pipelines should be automatically and continuously tested to evaluate their quality and usability. Also, manuscripts on software and methods development can be made available as preprints to accelerate their dissemination. Of course, these standards are not specific for coronavirus related research, but rather general points about bioinformatics software.

One major bottleneck hindering high software standards is the limited capacity of scientists to build versatile software, rather than prototypes. This might be improved by merging projects with similar or overlapping goals. However, this requires a central overview of newly developed tools and ongoing research projects and of how (future) products may fit together, e.g., in the form of a processing pipeline. Unfortunately, the life cycle of software in research is relatively short. Usually, scientific funding does not include the continuous maintenance of tools and pipelines so that developers are forced to move on to other projects and research grants.

The European Virus Bioinformatics Center curates a list of bioinformatics tools specifically for coronaviruses[29], some of which were presented in this review. ~~Neither this review nor the online list is complete, and in light of the rapid ongoing research, further tools will be developed.~~ Other initiatives are collecting relevant datasets (COVID-19 Data Portal[30]) or are supporting researchers by offering assistance with SARS-CoV-2 genome sequencing

[29] http://evbc.uni-jena.de/tools/coronavirus-tools/
[30] https://www.covid19dataportal.org/

(NFDI4Microbiota[31]). ELIXIR[32] provides a range of services to study SARS-CoV-2, in particular, the European Galaxy server for data-intensive research that provides access to scientific tools and training materials to guide users through COVID-19 data analysis. In addition, it is an encouraging development seeing researchers joining efforts in national and international initiatives to combat the ongoing pandemic. For example, researchers around the world are jointly reconstructing the molecular processes of the virus-host interactions to develop a COVID-19 Disease Map [126].

Key Points

- In light of the sheer amount of data, many fundamental questions in SARS-CoV-2 research can only be tackled with the help of bioinformatic tools.
- Bioinformatic analysis of SARS-CoV-2 data has the potential to track and trace SARS-CoV-2 sequence evolution and identify potential drug targets.
- All tools are free to use and~~freely~~ available online to rapidly advance SARS-CoV-2 research.

Availability

All presented tools are free to use and~~freely~~ available online, either through web applications or public code repositories. Licenses are given in Tab. 1. You can find a list of the presented tools and further tools on the EVBC website: http://evbc.uni-jena.de/tools/coronavirus-tools/

Acknowledgements

M.Hö. appreciates the support of the Joachim Herz Foundation by the add-on fellowship for interdisciplinary life science. P.L. acknowledges funding from the EpiPose project (European Union's SC1-PHE-CORONAVIRUS-2020 programme, H2020/101003688). Á.N.O.T. thanks Anthony Underwood and David Aanensen (Centre for Genomic Pathogen Surevillance, Hinxton, Cambridgeshire), as well as JT McCrone and Verity Hill (Rambaut Group, Edinburgh University) for contributions to the pangolin code. G.R.-P. thanks the CABANA Project for their support while conducting a research secondment at EMBL-EBI. We acknowledge the UniProt Consortium for the production of UniProt. GISAID acknowledgements can be found at this link: https://raw.githubusercontent.com/hCoV-2019/lineages/master/gisaid_acknowledgements.tsv

Funding

This work was supported by the Agencia Nacional de Promoción Científica y Tecnológica [PICT 2016-1327 and PICT 2017-2581 to M.C.]; the Biotechnology and Biological Sciences Research Council [BB/N018354/1 to A.A., BB/S020462/1 to A.B., and BB/P027849/1 to A.R. and G.R.-P.]; the Bundesministerium für Bildung und Forschung [5103388 to J.B., 13GW0096D and 13GW0423B to C.B., and 031L0176A to M.v.K.]; the Carl Zeiss Foundation [CZS 0563-2.8/738/2 to K.L.]; the Deutsche Forschungsgemeinschaft [FZT 118 to F.H., CRC 1076 to M.Hö., and SPP 1596 to M.M.]; the European Commission [H2020/777111 to S.S. and J.B., H2020/826078 to J.B., and ESF/14-BM-A55-0014/16 to L.K.,]; the European Molecular Biology Laboratory core funds to M.J.M.; the Fondation Innovations en Infectiologie [R12128CC to V.N.]; the Max Planck Society to D.K.; the Medical Research Council [MC_UU_1201412 to D.L.R. and J.B.S.]; the National Institutes of Health [GM080219 to S.H. and P.M., U24HG007822 to M.J.M., C.A., and N.R., U19 AI142777-02 to G.L., and Intramural Research Program of the National Library of Medicine to E.P.N.]; the Swiss Federal Government through the State Secretariat for Education, Research and Innovation to N.B. and N.R; the Velux Foundations [13154 to J.B.]; and the Wellcome Trust [203783/Z/16/Z to Á.N.O.T.].

Papers in preparation or submitted for publication should not be in the reference list.

References

[1] F. Wu, S. Zhao, B. Yu, et al. "A new coronavirus associated with human respiratory disease in China." *Nature* 579.7798 (2020), pp. 265–269.

[2] M. Hoffmann, H. Kleine-Weber, S. Schroeder, et al. "SARS-CoV-2 Cell Entry Depends on ACE2 and TMPRSS2 and Is Blocked by a Clinically Proven Protease Inhibitor". *Cell* 181.2 (2020), 271–280.e8.

[3] W. Li, M. J. Moore, N. Vasilieva, et al. "Angiotensin-converting enzyme 2 is a functional receptor for the SARS coronavirus". *Nature* 426.6965 (2003), pp. 450–454.

[4] I. Hamming, W. Timens, M. Bulthuis, et al. "Tnumber distribution of ACE2 protein, the functional receptor for SARS coronavirus. A first step in understanding SARS pathogenesis". *The Journal of Pathology* 203.2 (2004), pp. 631–637.

[5] A. C. Walls, Y.-J. Park, M. A. Tortorici, et al. "Structure, Function, and Antigenicity of the SARS-CoV-2 Spike Glycoprotein". *Cell* 181.2 (2020), 281–292.e6.

[6] H.-D. Klenk and W. Garten. "Host cell proteases controlling virus pathogenicity". *Trends Microbiol.* 2.2 (1994), pp. 39–43.

[7] D. A. Steinhauer. "Role of Hemagglutinin Cleavage for the Pathogenicity of Influenza Virus". *Virology* 258.1 (1999), pp. 1–20.

[8] J. K. Millet and G. R. Whittaker. "Host cell proteases: Critical determinants of coronavirus tropism and pathogenesis". *Virus Res.* 202 (2015), pp. 120–134.

[9] V. M. Corman, O. Landt, M. Kaiser, et al. "Detection of 2019 novel coronavirus (2019-nCoV) by real-time RT-PCR". *Eurosurveillance* 25.3 (2020).

[10] J. Quick. *nCoV-2019 sequencing protocol v1 (protocols.io.bbmuik6w)*. 2020.

[31] https://nfdi4microbiota.de/index.php/covid-19/
[32] https://elixir-europe.org/services/covid-19

[11] J. Quick, N. D. Grubaugh, S. T. Pullan, et al. "Multiplex PCR method for MinION and Illumina sequencing of Zika and other virus genomes directly from clinical samples". *Nat Protoc* 12.6 (2017), pp. 1261–1276.

[12] S. C. Moore, R. Penrice-Randal, M. Alruwaili, et al. "Amplicon based MinION sequencing of SARS-CoV-2 and metagenomic characterisation of nasopharyngeal swabs from patients with COVID-19". *medRxiv* (2020).

[13] S. Rampelli, E. Biagi, S. Turroni, et al. "Retrospective Search for SARS-CoV-2 in Human Faecal Metagenomes". *SSRN Electronic Journal* (2020).

[14] I. Sola, P. A. Mateos-Gomez, F. Almazan, et al. "RNA-RNA and RNA-protein interactions in coronavirus replication and transcription". *RNA Biology* 8.2 (2011), pp. 237–248.

[15] P. S. Masters. "Coronavirus genomic RNA packaging". *Virology* 537 (2019), pp. 198–207.

[16] S. J. Goebel, J. Taylor, and P. S. Masters. "The 3' cis-Acting Genomic Replication Element of the Severe Acute Respiratory Syndrome Coronavirus Can Function in the Murine Coronavirus Genome". *J. Virol.* 78.14 (2004), pp. 7846–7851.

[17] D. Yang and J. L. Leibowitz. "The structure and functions of coronavirus genomic 3' and 5' ends". *Virus Res.* 206 (2015), pp. 120–133.

[18] R. Madhugiri, N. Karl, D. Petersen, et al. "Structural and functional conservation of cis-acting RNA elements in coronavirus 5'-terminal genome regions". *Virology* 517 (2018), pp. 44–55.

[19] M. Hoffmann, M. T. Monaghan, and K. Reinert. "PriSeT: Efficient *De Novo* Primer Discovery". *bioRxiv* (2020).

[20] H. Li and R. Durbin. "Fast and accurate short read alignment with Burrows-Wheeler transform." *Bioinformatics* 25.14 (2009), pp. 1754–1760.

[21] A. McKenna, M. Hanna, E. Banks, et al. "The Genome Analysis Toolkit: a MapReduce framework for analyzing next-generation DNA sequencing data." *Genome Res* 20.9 (2010), pp. 1297–1303.

[22] J. Köster and S. Rahmann. "Snakemake—a scalable bioinformatics workflow engine". *Bioinformatics* 28.19 (2012), pp. 2520–2522.

[23] B. Grüning, R. Dale, A. Sjödin, et al. "Bioconda: sustainable and comprehensive software distribution for the life sciences." *Nat Methods* 15.7 (2018), pp. 475–476.

[24] P. D. Tommaso, M. Chatzou, E. W. Floden, et al. "Nextflow enables reproducible computational workflows". *Nat Biotechnol* 35.4 (2017), pp. 316–319.

[25] A. A. Schäffer, E. L. Hatcher, L. Yankie, et al. "VADR: validation and annotation of virus sequence submissions to GenBank". *BMC Bioinformatics* 21.1 (2020).

[26] N. A. O'Leary, M. W. Wright, J. R. Brister, et al. "Reference sequence (RefSeq) database at NCBI: current status, taxonomic expansion, and functional annotation." *Nucleic acids research* 44 (D1 2016), pp. D733–D745.

[27] S. Posada-Céspedes, D. Seifert, I. Topolsky, et al. "V-pipe: a computational pipeline for assessing viral genetic diversity from high-throughput sequencing data". *bioRxiv* (2020).

[28] J. L. Waner. "Mixed viral infections: detection and management." *Clin Microbiol Rev* 7.2 (1994), pp. 143–151.

[29] N. Kumar, S. Sharma, S. Barua, et al. "Virological and Immunological Outcomes of Coinfections". *Clin Microbiol Rev* 31.4 (2018).

[30] D. Lin, L. Liu, M. Zhang, et al. "Co-infections of SARS-CoV-2 with multiple common respiratory pathogens in infected patients". *Sci China Life Sci* 63.4 (2020), pp. 606–609.

[31] A. L. Mitchell, A. Almeida, M. Beracochea, et al. "MGnify: the microbiome analysis resource in 2020". *Nucleic Acids Res* (2019).

[32] D. Li, C.-M. Liu, R. Luo, et al. "MEGAHIT: an ultra-fast single-node solution for large and complex metagenomics assembly via succinct de Bruijn graph". *Bioinformatics* 31.10 (2015), pp. 1674–1676.

[33] Z. Gu, L. Gu, R. Eils, et al. "circlize implements and enhances circular visualization in R". *Bioinformatics* 30.19 (2014), pp. 2811–2812.

[34] A. Ehlers, J. Osborne, S. Slack, et al. "Poxvirus Orthologous Clusters (POCs)". *Bioinformatics* 18.11 (2002), pp. 1544–1545.

[35] R. Brodie, A. J. Smith, R. L. Roper, et al. "Base-By-Base: Single nucleotide-level analysis of whole viral genome alignments". *BMC Bioinformatics* 5.1 (2004), p. 96.

[36] W. Hillary, S.-H. Lin, and C. Upton. "Base-By-Base version 2: single nucleotide-level analysis of whole viral genome alignments". *Microb Inf Exp* 1.1 (2011), p. 2.

[37] S.-L. Tu, J. Staheli, C. McClay, et al. "Base-By-Base Version 3: New Comparative Tools for Large Virus Genomes". *Viruses* 10.11 (2018), p. 637.

[38] C. Upton, D. Hogg, D. Perrin, et al. "Viral genome organizer: a system for analyzing complete viral genomes". *Virus Res* 70.1-2 (2000), pp. 55–64.

[39] V. Tcherepanov, A. Ehlers, and C. Upton. "Genome Annotation Transfer Utility (GATU): rapid annotation of viral genomes using a closely related reference genome". *BMC Genomics* 7.1 (2006).

[40] P. J. K. Libin, K. Deforche, A. B. Abecasis, et al. "VIRULIGN: fast codon-correct alignment and annotation of viral genomes." *Bioinformatics* 35.10 (2019), pp. 1763–1765.

[41] L. Cuypers, G. Li, C. Neumann-Haefelin, et al. "Mapping the genomic diversity of HCV subtypes 1a and 1b: Implications of structural and immunological constraints for vaccine and drug development." *Virus Evol* 2.2 (2016), vew024.

[42] S. Ngcapu, K. Theys, P. Libin, et al. "Characterization of Nucleoside Reverse Transcriptase Inhibitor-Associated Mutations in the RNase H Region of HIV-1 Subtype C Infected Individuals." *Viruses* 9.11 (2017).

[43] A.-C. Pineda-Peña, M. Pingarilho, G. Li, et al. "Drivers of HIV-1 transmission: The Portuguese case." *PLoS One* 14.9 (2019), e0218226.

[44] K. Theys, P. Libin, K. Dallmeier, et al. "Zika genomics urgently need standardized and curated reference sequences." *PLoS Pathog* 13.9 (2017), e1006528.

[45] A. Rambaut, E. C. Holmes, V. Hill, et al. "A dynamic nomenclature proposal for SARS-CoV-2 to assist genomic epidemiology". *bioRxiv* (2020).

[46] L. Cuypers, P. Libin, Y. Schrooten, et al. "Exploring resistance pathways for first-generation NS3/4A protease inhibitors boceprevir and telaprevir using Bayesian network learning." *Infect Genet Evol* 53 (2017), pp. 15–23.

[47] P. Libin, E. V. Eynden, F. Incardona, et al. "PhyloGeoTool: interactively exploring large phylogenies in an epidemiological context". *Bioinformatics* 33.24 (2017). Ed. by J. Kelso, pp. 3993–3995.

[48] P. Libin, G. Beheydt, K. Deforche, et al. "RegaDB: community-driven data management and analysis for infectious diseases." *Bioinformatics* 29.11 (2013), pp. 1477–1480.

[49] I. Kalvari, J. Argasinska, N. Quinones-Olvera, et al. "Rfam 13.0: shifting to a genome-centric resource for non-coding RNA families." *Nucleic Acids Res* 46.D1 (2018), pp. D335–D342.

[50] E. P. Nawrocki and S. R. Eddy. “Infernal 1.1: 100-fold faster RNA homology searches.” *Bioinformatics* 29.22 (2013), pp. 2933–2935.

[51] UniProt Consortium. “UniProt: a worldwide hub of protein knowledge.” *Nucleic Acids Res* 47.D1 (2019), pp. D506–D515.

[52] X. Watkins, L. J. Garcia, S. Pundir, et al. “ProtVista: visualization of protein sequence annotations.” *Bioinformatics* 33.13 (2017), pp. 2040–2041.

[53] S. El-Gebali, J. Mistry, A. Bateman, et al. “The Pfam protein families database in 2019”. *Nucleic Acids Res.* 47.D1 (2018), pp. D427–D432.

[54] S. R. Eddy. “Accelerated Profile HMM Searches”. *PLoS Comput Biol* 7.10 (2011). Ed. by W. R. Pearson, e1002195.

[55] E. M. Volz, S. L. K. Pond, M. J. Ward, et al. “Phylodynamics of Infectious Disease Epidemics”. *Genetics* 183.4 (2009), pp. 1421–1430.

[56] A. Torneri, P. Libin, J. Vanderlocht, et al. “A prospect on the use of antiviral drugs to control local outbreaks of COVID-19.” *BMC Med* 18 (1 2020), p. 191.

[57] Y. Jin, H. Yang, W. Ji, et al. “Virology, Epidemiology, Pathogenesis, and Control of COVID-19.” *Viruses* 12 (4 2020).

[58] S. Solis-Reyes, M. Avino, A. Poon, et al. “An open-source k-mer based machine learning tool for fast and accurate subtyping of HIV-1 genomes.” *PLoS One* 13.11 (2018), e0206409.

[59] W. Chang, J. Cheng, J. Allaire, et al. *shiny: Web Application Framework for R. R package version 1.4.0.2.* 2020.

[60] J. Hadfield, C. Megill, S. M. Bell, et al. “Nextstrain: real-time tracking of pathogen evolution”. *Bioinformatics* 34.23 (2018), pp. 4121–4123.

[61] S. Elbe and G. Buckland-Merrett. “Data, disease and diplomacy: GISAID’s innovative contribution to global health”. *Global Challenges* 1.1 (2017), pp. 33–46.

[62] M. N. Wright and A. Ziegler. “ranger: A Fast Implementation of Random Forests for High Dimensional Data in C++ and R”. *J Stat Softw* 77.1 (2017).

[63] L. Breiman. “Random Forests”. *Mach Learn* 45.1 (2001), pp. 5–32.

[64] K. Katoh and D. M. Standley. “MAFFT Multiple Sequence Alignment Software Version 7: Improvements in Performance and Usability”. *Mol. Biol. Evol.* 30.4 (2013), pp. 772–780.

[65] B. Q. Minh, M. A. T. Nguyen, and A. von Haeseler. “Ultrafast approximation for phylogenetic bootstrap.” *Mol Biol Evol* 30.5 (2013), pp. 1188–1195.

[66] B. Q. Minh, H. A. Schmidt, O. Chernomor, et al. “IQ-TREE 2: New Models and Efficient Methods for Phylogenetic Inference in the Genomic Era”. *Mol Biol Evol* 37.5 (2020). Ed. by E. Teeling, pp. 1530–1534.

[67] B. T. Grenfell. “Unifying the Epidemiological and Evolutionary Dynamics of Pathogens”. *Science* 303.5656 (2004), pp. 327–332.

[68] R. Bouckaert, T. G. Vaughan, J. Barido-Sottani, et al. “BEAST 2.5: An advanced software platform for Bayesian evolutionary analysis”. *PLoS Comput Biol* 15.4 (2019). Ed. by M. Pertea, e1006650.

[69] T. Stadler, D. Kuhnert, S. Bonhoeffer, et al. “Birth-death skyline plot reveals temporal changes of epidemic spread in HIV and hepatitis C virus (HCV)”. *Proc Natl Acad Sci USA* 110.1 (2013), pp. 228–233.

[70] D. Kühnert, T. Stadler, T. G. Vaughan, et al. “The Birth-Death SIR model: simultaneous reconstruction of evolutionary history and epidemiological dynamics from viral sequences”. in revision. 2013.

[71] D. Kühnert, T. Stadler, T. G. Vaughan, et al. “Phylodynamics with Migration: A Computational Framework to Quantify Population Structure from Genomic Data”. *Mol Biol Evol* 33.8 (2016), pp. 2102–2116.

[72] T. G. Vaughan, D. Welch, A. J. Drummond, et al. “Inferring Ancestral Recombination Graphs from Bacterial Genomic Data”. *Genetics* 205.2 (2017), pp. 857–870.

[73] N. De Maio, C. J. Worby, D. J. Wilson, et al. “Bayesian reconstruction of transmission within outbreaks using genomic variants”. *PLoS Comput Biol* 14.4 (2018). Ed. by K. Koelle, e1006117.

[74] E. M. Volz and I. Siveroni. “Bayesian phylodynamic inference with complex models”. *PLoS Comput Biol* 14.11 (2018). Ed. by A. E. Darling, e1006546.

[75] T. G. Vaughan, G. E. Leventhal, D. A. Rasmussen, et al. “Estimating Epidemic Incidence and Prevalence from Genomic Data”. *Mol Biol Evol* 36.8 (2019). Ed. by D. Falush, pp. 1804–1816.

[76] S. Reimering, S. Muñoz, and A. C. McHardy. “Phylogeographic reconstruction using air transportation data and its application to the 2009 H1N1 influenza A pandemic”. *PLoS Comput Biol* 16.2 (2020). Ed. by C. Viboud, e1007101.

[77] D. Brockmann and D. Helbing. “The Hidden Geometry of Complex, Network-Driven Contagion Phenomena”. *Science* 342.6164 (2013), pp. 1337–1342.

[78] S. Hoops, S. Sahle, R. Gauges, et al. “COPASI—a complex pathway simulator”. *Bioinformatics* 22.24 (2006), pp. 3067–3074.

[79] L. G. Gebhard, S. B. Kaufman, and A. V. Gamarnik. “Novel ATP-independent RNA annealing activity of the dengue virus NS3 helicase”. *PLoS One* 7.4 (2012).

[80] R. B. Tunnicliffe, G. M. Hautbergue, S. A. Wilson, et al. “Competitive and cooperative interactions mediate RNA transfer from herpesvirus saimiri ORF57 to the mammalian export adaptor ALYREF”. *PLoS Pathog* 10.2 (2014).

[81] S. Sheppard and D. Dikicioglu. “Dynamic modelling of the killing mechanism of action by virus-infected yeasts”. *J R Soc Interface* 16.152 (2019), p. 20190064.

[82] F. Tapia, T. Laske, M. A. Wasik, et al. “Production of defective interfering particles of influenza A virus in parallel continuous cultures at two residence times–insights from qPCR measurements and viral dynamics modeling”. *Front Bioeng Biotechnol* 7 (2019), p. 275.

[83] L. U. Aguilera and J. Rodríguez-González. “Modeling the effect of tat inhibitors on HIV latency”. *J Theor Biol* 473 (2019), pp. 20–27.

[84] J. K. Barry. “Mathematical Modelling of the HIV Life Cycle: Identifying Optimal Treatment Strategies”. PhD thesis. University of Greifswald, 2018.

[85] S. Kaliamurthi, G. Selvaraj, A. C. Kaushik, et al. “Designing of CD8+ and CD8+-overlapped CD4+ epitope vaccine by targeting late and early proteins of human papillomavirus”. *Biol: Targets Ther* 12 (2018), p. 107.

[86] A. Akgül, S. H. A. Khoshnaw, and W. H. Mohammed. “Mathematical Model for the Ebola Virus Disease”. *J Adv Phys* 7.2 (2018), pp. 190–198.

[87] C. Zimmer, S. I. Leuba, T. Cohen, et al. “Accurate quantification of uncertainty in epidemic parameter estimates and predictions using stochastic compartmental models”. *Stat Methods Med Res* 28.12 (2018), pp. 3591–3608.

[88] C. Zimmer, R. Yaesoubi, and T. Cohen. “A Likelihood Approach for Real-Time Calibration of Stochastic Compartmental Epidemic Models.” *PLoS Comput Biol* 13.1 (2017), e1005257.

[89] H. V. Westerhoff and A. N. Kolodkin. “Advice from a systems-biology model of the corona epidemics”. *npj Syst. Biol. Appl.* 6.1 (2020).

[90] W. O. Kermack and A. G. McKendrick. “A contribution to the mathematical theory of epidemics”. *Proc R Soc A* 115.772 (1927), pp. 700–721.

[91] L. R. Lopez and X. Rodo. “A modified SEIR model to predict the COVID-19 outbreak in Spain and Italy: simulating control scenarios and multi-scale epidemics”. *medRxiv* (2020).

[92] S. Khailaie, T. Mitra, A. Bandyopadhyay, et al. “Estimate of the development of the epidemic reproduction number Rt from Coronavirus SARS-CoV-2 case data and implications for political measures based on prognostics”. *medRxiv* (2020).

[93] J. B. Singer, E. C. Thomson, J. McLauchlan, et al. “GLUE: a flexible software system for virus sequence data”. *BMC Bioinf* 19.1 (2018).

[94] Y. Shu and J. McCauley. “GISAID: Global initiative on sharing all influenza data – from vision to reality”. *Eurosurveillance* 22.13 (2017).

[95] A. Stamatakis. “RAxML version 8: a tool for phylogenetic analysis and post-analysis of large phylogenies”. *Bioinformatics* 30.9 (2014), pp. 1312–1313.

[96] Z. Yang. “PAML 4: phylogenetic analysis by maximum likelihood.” *Mol Biol Evol* 24.8 (2007), pp. 1586–1591.

[97] L. Velazquez-Salinas, S. Zarate, S. Eberl, et al. “Positive selection of ORF3a and ORF8 genes drives the evolution of SARS-CoV-2 during the 2020 COVID-19 pandemic”. *bioRxiv* (2020).

[98] B. Korber, W. Fischer, S. Gnanakaran, et al. “Spike mutation pipeline reveals the emergence of a more transmissible form of SARS-CoV-2”. *bioRxiv* (2020).

[99] M. Hölzer and M. Marz. “PoSeiDon: a Nextflow pipeline for the detection of evolutionary recombination events and positive selection”. *bioRxiv* (2020).

[100] G. Schneider and U. Fechner. “Computer-based *de novo* design of drug-like molecules”. *Nat. Rev. Drug Discovery* 4.8 (2005), pp. 649–663.

[101] I. Kapetanovic. “Computer-aided drug discovery and development (CADDD): In silico-chemico-biological approach”. *Chem. Biol. Interact.* 171.2 (2008), pp. 165–176.

[102] T. Guirimand, S. Delmotte, and V. Navratil. “VirHostNet 2.0: surfing on the web of virus/host molecular interactions data”. *Nucleic Acids Res* 43.D1 (2014), pp. D583–D587.

[103] D. E. Gordon, G. M. Jang, M. Bouhaddou, et al. “A SARS-CoV-2 protein interaction map reveals targets for drug repurposing”. *Nature* (2020).

[104] S.-A. Sansone, P. McQuilton, et al. “FAIRsharing as a community approach to standards, repositories and policies”. *Nat Biotechnol* 37.4 (2019), pp. 358–367.

[105] V. Navratil, L. Lionnard, S. Longhi, et al. “The severe acute respiratory syndrome coronavirus 2 (SARS-CoV-2) envelope (E) protein harbors a conserved BH3-like sequence”. *bioRxiv* (2020).

[106] R. Martin, H. F. Löchel, M. Welzel, et al. “CORDITE: The Curated CORona Drug InTEractions Database for SARS-CoV-2”. *iScience* 23.7 (2020), p. 101297.

[107] S. Sadegh, J. Matschinske, D. B. Blumenthal, et al. “Exploring the SARS-CoV-2 virus-host-drug interactome for drug repurposing”. *Nat Commun* 11.1 (2020).

[108] S. Pfefferle, J. Schöpf, M. Kögl, et al. “The SARS-Coronavirus-Host Interactome: Identification of Cyclophilins as Target for Pan-Coronavirus Inhibitors”. *PLoS Pathog* 7.10 (2011). Ed. by M. R. Denison, e1002331.

[109] M. Kotlyar, C. Pastrello, Z. Malik, et al. “IID 2018 update: context-specific physical protein–protein interactions in human, model organisms and domesticated species”. *Nucleic Acids Res* 47.D1 (2018), pp. D581–D589.

[110] D. Mendez, A. Gaulton, A. P. Bento, et al. “ChEMBL: towards direct deposition of bioassay data”. *Nucleic Acids Res* 47.D1 (2018), pp. D930–D940.

[111] D. S. Wishart, Y. D. Feunang, A. C. Guo, et al. “DrugBank 5.0: a major update to the DrugBank database for 2018”. *Nucleic Acids Res* 46.D1 (2017), pp. D1074–D1082.

[112] O. Ursu, J. Holmes, C. G. Bologa, et al. “DrugCentral 2018: an update”. *Nucleic Acids Res* 47.D1 (2018), pp. D963–D970.

[113] Y. Wang, S. Zhang, F. Li, et al. “Therapeutic target database 2020: enriched resource for facilitating research and early development of targeted therapeutics”. *Nucleic Acids Res* (2019).

[114] D. A. Young, J. A. DeQuach, and K. L. Christman. “Human cardiomyogenesis and the need for systems biology analysis”. *Wiley Interdiscip Rev Syst Biol Med* 3.6 (2010), pp. 666–680.

[115] M. K. Gilson, T. Liu, M. Baitaluk, et al. “BindingDB in 2015: A public database for medicinal chemistry, computational chemistry and systems pharmacology”. *Nucleic Acids Res* 44.D1 (2015), pp. D1045–D1053.

[116] G. Lasso, S. V. Mayer, E. R. Winkelmann, et al. “A Structure-Informed Atlas of Human-Virus Interactions”. *Cell* 178.6 (2019), 1526–1541.e16.

[117] J. I. Garzón, L. Deng, D. Murray, et al. “A computational interactome and functional annotation for the human proteome”. *eLife* 5 (2016).

[118] Q. C. Zhang, D. Petrey, L. Deng, et al. “Structure-based prediction of protein–protein interactions on a genome-wide scale”. *Nature* 490.7421 (2012), pp. 556–560.

[119] R. D. Finn, P. Coggill, R. Y. Eberhardt, et al. “The Pfam protein families database: towards a more sustainable future”. *Nucleic Acids Res* 44.D1 (2015), pp. D279–D285.

[120] Gene Ontology Consortium. “Expansion of the Gene Ontology knowledgebase and resources”. *Nucleic Acids Res* 45.D1 (2017), pp. D331–D338.

[121] A Bairoch. “The ENZYME database in 2000.” *Nucleic Acids Res* 28.1 (2000), pp. 304–305.

[122] A. Subramanian, P. Tamayo, V. K. Mootha, et al. “Gene set enrichment analysis: A knowledge-based approach for interpreting genome-wide expression profiles”. *Proc Natl Acad Sci USA* 102.43 (2005), pp. 15545–15550.

[123] L. M. Schriml, M. Chuvochina, N. Davies, et al. “COVID-19 pandemic reveals the peril of ignoring metadata standards”. *Sci Data* 7.1 (2020).

[124] C. Boettiger. “An introduction to Docker for reproducible research”. *ACM SIGOPS Operating Systems Review* 49.1 (2015), pp. 71–79.

[125] P. A. Ewels, A. Peltzer, S. Fillinger, et al. “The nf-core framework for community-curated bioinformatics pipelines.” *Nat Biotechnol* 38 (3 2020), pp. 276–278.

[126] M. Ostaszewski, A. Mazein, M. E. Gillespie, et al. “COVID-19 Disease Map, building a computational repository of SARS-CoV-2 virus-host interaction mechanisms”. *Sci Data* 7.1 (2020).
